# RNA-seq Transcriptome Response of Flax (*Linum usitatissimum* L.) to the Pathogenic Fungus *Fusarium oxysporum* f. sp. *lini*

**DOI:** 10.3389/fpls.2016.01766

**Published:** 2016-11-24

**Authors:** Leonardo Galindo-González, Michael K. Deyholos

**Affiliations:** ^1^Department of Biological Sciences, University of Alberta, EdmontonAB, Canada; ^2^IK Barber School of Arts and Sciences, University of British Columbia, KelownaBC, Canada

**Keywords:** flax, transcriptome, *Linum usitatissimum*, RNA-seq, *Fusarium oxysporum*

## Abstract

*Fusarium oxysporum* f. sp. *lini is* a hemibiotrophic fungus that causes wilt in flax. Along with rust, fusarium wilt has become an important factor in flax production worldwide. Resistant flax cultivars have been used to manage the disease, but the resistance varies, depending on the interactions between specific cultivars and isolates of the pathogen. This interaction has a strong molecular basis, but no genomic information is available on how the plant responds to attempted infection, to inform breeding programs on potential candidate genes to evaluate or improve resistance across cultivars. In the current study, disease progression in two flax cultivars [Crop Development Center (CDC) Bethune and Lutea], showed earlier disease symptoms and higher susceptibility in the later cultivar. Chitinase gene expression was also divergent and demonstrated and earlier molecular response in Lutea. The most resistant cultivar (CDC Bethune) was used for a full RNA-seq transcriptome study through a time course at 2, 4, 8, and 18 days post-inoculation (DPI). While over 100 genes were significantly differentially expressed at both 4 and 8 DPI, the broadest deployment of plant defense responses was evident at 18 DPI with transcripts of more than 1,000 genes responding to the treatment. These genes evidenced a reception and transduction of pathogen signals, a large transcriptional reprogramming, induction of hormone signaling, activation of pathogenesis-related genes, and changes in secondary metabolism. Among these, several key genes that consistently appear in studies of plant-pathogen interactions, had increased transcript abundance in our study, and constitute suitable candidates for resistance breeding programs. These included: an induced *R*PMI-induced protein kinase; transcription factors *WRKY3, WRKY70, WRKY75, MYB113*, and *MYB108*; the ethylene response factors *ERF1* and *ERF14*; two genes involved in auxin/glucosinolate precursor synthesis (*CYP79B2* and *CYP79B3*); the flavonoid-related enzymes chalcone synthase, dihydroflavonol reductase and multiple anthocyanidin synthases; and a peroxidase implicated in lignin formation (*PRX52*). Additionally, regulation of some genes indicated potential pathogen manipulation to facilitate infection; these included four disease resistance proteins that were repressed, indole acetic acid amido/amino hydrolases which were upregulated, activated expansins and glucanases, amino acid transporters and aquaporins, and finally, repression of major latex proteins.

## Introduction

Flax (*Linum usitatissimum*) is an important crop for the production of fiber, oil, and nutraceuticals (Vaisey-Genser and Morris, 2003). Among flax diseases caused by fungal pathogens, fusarium wilt, caused by *Fusarium oxysporum* f. sp. *lini* (*Foln*) has been an important factor in limiting yield of this plant. Fusarium wilt was identified as a major flax disease problem in North America at the beginning of the 20th century (Rashid, 2003). *Fusarium* is a genus of filamentous, seed, and soil-borne ascomycetes with numerous pathogenic members that have been reported to cause disease in over 100 major crop species worldwide (Ma et al., 2013). Besides wilt disease, it can also produce rots, blights, and cankers through invasive growth and the production of mycotoxins, using mainly a hemibiotrophic infection strategy (Ma et al., 2013). Infection occurs through the roots, invading the water-conducting tissues, which impairs water transport and results in wilting, necrosis, and chlorosis of aerial parts (Rashid, 2003; Ma et al., 2013). *Fusarium oxysporum* can persist in the soil for 5–10 years (Rashid, 2003), which allows recurrent infections if soil and residues are not treated and if no crop rotation is implemented. While the generation of fusarium-resistant cultivars worldwide has reduced the impact of the pathogen, there is a wide range of susceptibility among varieties, dependent in part on the specific fungal isolates/races involved in infection (Kroes et al., 1999).

Previous studies of interactions between flax and fusarium have focused on disease symptomatology (Kroes et al., 1998b), physiology and the fungal colonization process (Kroes et al., 1998a; Olivain et al., 2003; Hano et al., 2008). At the molecular level, the gene for gene relationship, where a resistance gene (*R*) in the plant interacts with a pathogen effector (initially designated as avirulence gene), was initially described for the flax-rust (*Melampsora lini*) interaction (Flor, 1956). These resistance genes in flax presented great allelic variability (Ellis et al., 1999), which is in agreement with continuous evolution to create new determinants that can recognize variants of the pathogens effectors (Ravensdale et al., 2011). However, while extensive work has been done on the flax-rust interaction at the level of *R-*genes and pathogen effectors (reviewed Ravensdale et al., 2011), other whole genome responses involving the rest of the signaling and defense processes are largely unexplored. In the meantime, some specific molecular and metabolic responses have been studied for the flax-fusarium interaction. To date, studies that have explored multiple responses simultaneously include: (i) a study of the responses to *F. oxysporum* infection using a cDNA subtraction methodology between *F. oxysporum*-infected and control plants. The study resulted in the identification of 47 genes involved in defense signaling and response, stress response and changes in primary an secondary metabolism (Kostyn et al., 2012); and (ii) the study of molecular events associated with cell death after inoculation with *F. oxysporum*, which included the production of reactive oxygen species and associated lipid peroxidation, enzyme-mediated DNA degradation, and induction of phenylpropanoid metabolism (Hano et al., 2008). Other studies have addressed responses of specific genes or gene families upon *F. oxysporum* attack based on the knowledge of the general responses in plant-pathogen interactions; such studies display different stages and mechanisms of defense. For example, a study of changes in polyamine gene expression and polyamine metabolites in response to *F. oxysporum* and *F. culmorum* (Wojtasik et al., 2015), was explored due to the regulation of arginine decarboxylase in the aforementioned cDNA subtraction study. Others have used transgenics to modulate secondary metabolites (carotenoid and flavonoid biosynthesis) to increase resistance of flax to the pathogen, using the antioxidant action of these compounds against the reactive oxygen species produced upon infection (Lorenc-Kukuła et al., 2007, 2009; Boba et al., 2011). Likewise, studies of the behavior of cell wall modification genes and the metabolism of lignin production upon fusarium inoculation showed that some genes are modulated to increase resistance while others might be manipulated by the pathogen (Hano et al., 2006; Wojtasik et al., 2011, 2016). Techniques that have been applied to study these processes include: transformation (Wróbel-Kwiatkowska et al., 2004; Lorenc-Kukuła et al., 2007, 2009; Boba et al., 2011), tissue culture (Rutkowska-Krause et al., 2003), and QTL analysis (Spielmeyer et al., 1998). To date there have been no transcriptome-scale studies of the response of flax to *F. oxysporum* f. sp. *lini*, which limits information that can be used to further breeding improvements.

RNA-seq studies of plant responses to fungal pathogens have been performed in numerous pathosystems including: lettuce infected by *Botrytis cinerea* (De Cremer et al., 2013), *Arabidopsis thaliana* after treatment with *Pseudomonas syringae* (Howard et al., 2013) chrysanthemum leaf after infection with *Alternaria tenuissima* (Li et al., 2014), a wheat resistant variety affected by *Fusarium graminearum* (Xiao et al., 2013), and the early infection of peach leaves by *Xanthomonas arboricola* (Socquet-Juglard et al., 2013). Specifically for the interaction with *Fusarium oxysporum*, studies were made in *A. thaliana* (Zhu et al., 2013b), banana roots (Li et al., 2012, 2013; Wang et al., 2012b), and cabbage (Xing et al., 2016); and additional research was made on the interaction of *F. graminearum* with wheat (Xiao et al., 2013; Erayman et al., 2015). All of these global studies have shown a general picture of plant defenses where the processes of signal transduction, ion status, oxidative and detoxification control, transcriptional regulation, pathogen-related gene response, hormone modulation, activation of secondary metabolism (e.g., phenylpropanoids), and regulation of transporters, are central to the plant defense.

The sequencing and annotation of the flax genome (Wang et al., 2012a) has unlocked new genomic tools that can be used for whole genome scale studies. The flax genome sequence was based on the cultivar CDC (Crop Development Center) Bethune, which is a highly inbred, elite oilseed cultivar widely grown in Canada (Rowland et al., 2002). Furthermore, CDC Bethune has been classified as moderately resistant to fusarium wilt (Rowland et al., 2002), although other studies have found higher levels of susceptibility (Diederichsen et al., 2008), which supports the need to investigate the resistance mechanisms of this elite cultivar.

Here, we present a multi-level study of the progression of *Foln*-induced responses in CDC Bethune contrasted with Lutea, which is an exemplar of a less-resistant cultivar. The relative susceptibility of the two cultivars was demonstrated by monitoring disease symptoms following inoculation with *F. oxysporum* f. sp. *lini*, and by measuring changes in chitinase transcript expression as a marker of defense responses. Finally, we conducted RNA-seq analysis on CDC Bethune, following infection by *F. oxysporum* f. sp. *lini*. Besides the deployment of a full defense response from the plant at the end of the evaluated time course, several genes had unexpected patterns of regulation which supported growth, weakening of the cell wall, and favored fungal penetration, and may be indicative of partial manipulation of host genes by the pathogen. The genes identified can be used to inform breeding programs and improve understanding of molecular mechanisms underlying fusarium resistance.

## Materials and Methods

### Plant Material

**FIGURE 1:**
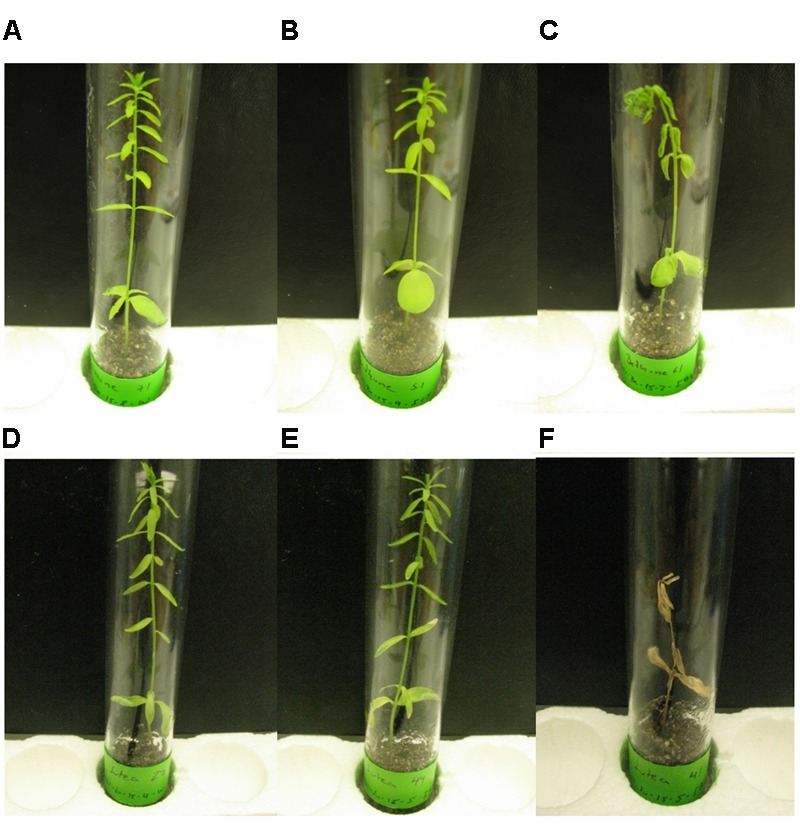
**Disease symptoms 22 DPI in flax cultivars CDC Bethune (A–C)** and Lutea **(D–F)**. **(A)** and **(D)**: Control plants treated with water. **(B)** and **(E)**: Plants inoculated with isolate #65. **(C)** and **(F)**: Plants inoculated with isolate #81.

Seeds from flax cultivars CDC Bethune and Lutea were grown according to the protocol of Kroes et al. (1998b) with some modifications: sterilized seeds from each cultivar were grown in sterile 25mm × 200 mm glass tubes filled with 5 mL of 10% Murashige-Skoog solution (MS basal medium Sigma–Aldrich, St. Louis, MO, USA) pH 5.8 and 2 g of vermiculite (**Figure 1**). Tubes were placed in a growth chamber at 22°C with 16 h day/8 h night (light intensity = 167 μMol).

### Pathogen

*Fusarium oxysporum* f. sp. *lini* isolates (#65 and #81) were kindly provided in potato dextrose agar (PDA) by Khalid Rashid (Agriculture and Agri-Food Canada, Morden, MB, Canada). Isolate #65 is from the Indian Head Saskatchewan flax nursery, and isolate #81 was obtained from a farmer’s field in Treherne, MB, Canada. We grew *F. oxysporum* isolates in PDA (39 g/L) plates at 21°C under 12 h dark/12 h light cycles. Cultures were started on three consecutive days, and viability was assessed by counting percentage of germinated spores at 1, 4, 8, and 24 h when the initiated cultures reached 13, 14, and 15 days to select the best culture for inoculation. Spores (a mix of macro and microconidia) from isolates were harvested after flooding the plate with 15 mL of sterile water and a sterile inoculation loop was used to detach the mycelium/spores from the surface of the media. Spore count was performed using a haemocytometer. Spore suspensions were diluted to 10^5^ spores mL^-1^ to perform the inoculations.

### Comparison of Cultivar Response

Plants grown in test tubes (described above) until cotyledon expansion, were either inoculated with 1 mL of 10^5^ spores mL^-1^ of the fungal isolates, or with 1 mL of sterile water (control) directly on the surface of the vermiculite under sterile conditions. Disease symptoms and shoot length were recorded at 1, 8, and 22 days post-inoculation (DPI) for 7–10 plants from each treatment (control, isolates #81 and #65) in each cultivar. Plants were removed from vermiculite and roots were cleaned with sterile water and dried. Sections of 3–5 cm from root tips were taken from four plants of each treatment for microscopy and fungal isolation from infected plants (see below). Entire seedlings were placed in 2 mL tubes and flash-frozen in liquid nitrogen for further processing.

### Fungal Isolation from Infected Plants

To confirm that symptoms were a result of the fungal infection, *F. oxysporum* f. sp. *lini* was reisolated from the plant roots. Collected root tip sections (3–5 cm) were surface sterilized in 10% sodium hypochlorite for 30 s and then rinsed three times in sterile distilled water. Root sections were further cut into 3–5 mm sections and air-dried on Whatman paper. Four to six of these root sections were transferred to sterile Komada medium (Leslie and Summerell, 2006) and grown for 7 days at 22°C under 8 h dark/16 h light cycles. Plates were examined for growth, and colonies were subcultured in PDA for 14 additional days at 21°C under 12 h dark/12 h light cycles.

### Microscopy

To examine fungal penetration of plant tissues, we collected root sections of 3–5 mm in length that were fixed in FAA (3.7% Formaldehyde, 5% Acetic acid- 50% Alcohol), then dehydrated in an ethanol series (50 and 70%) and embedded in paraffin blocks using the TISSUE TEK II embedding center (Sakura, Torrance, CA, USA). Sections of 8 and 12 μm were cut from the blocks using a RM2125 microtome (Leica, Wetzlar, Germany), and stained with 0.5% (w/v) Toluidine Blue. Sections were observed with a Leica DMRXA microscope (Meyer Instruments, Houston, TX, USA), photographed with the incorporated QI Click digital camera and captured using the Q Capture Pro 7 software (Q Imaging, Surrey, BC, Canada).

### RNA Extraction and cDNA Synthesis

Entire plants collected from the time course were used for RNA extraction and cDNA synthesis to evaluate gene expression. Tissue was ground in 2 mL collection tubes with a 5.5 mm stainless steel bead, using a Mixer Mill MM 301 (Retsch, Haan, Germany). RNA was extracted using the RNeasy Plant Mini Kit (QIAGEN, Venlo, Netherlands), followed by a DNAse I treatment (Ambion-Life Technologies, Carlsbad, CA, USA). RNA quality was checked with a 2100 Bioanalyzer (Agilent, Mississauga, ON, Canada), and cDNA was synthesized with 250 ng of RNA using the RevertAid H Minus Reverse transcriptase using oligo dT (18) (Thermo Scientific, Waltham, MA, USA). Presence of contaminating genomic DNA was tested by PCR analysis using pectinesterase gene primers (Supplementary Table S1), which give two distinct bands of 123 bp and 848 bp for cDNA and genomic DNA, respectively.

### Quantitative Reverse Transcription PCR (qRT-PCR)

To test the defense response of the two cultivars, selected flax chitinases were chosen as orthologs of genes previously characterized in *A. thaliana* (Passarinho and de Vries, 2002). Four chitinases (chitinase-like CTL2, 4, 10, and 11) from Glycosyl Hydrolase family 19 (GH19) were selected to test the response to the pathogen (Mokshina et al., 2014). To select reference genes, we tested primers from six genes for stability upon our treatments from a list of 13 genes previously published as normalizers in qRT-PCR experiments in flax (Huis et al., 2010): Elongation factor 1-α (EF1A), Eukaryotic translation initiation factor 3E (ETIF3E), Eukaryotic translation factor 5A (ETIF5A), Glyceraldehyde 3-phosphate dehydrogenase (GAPDH), Ubiquitin (UBI), and Ubiquitin extension protein (UBI2; Supplementary Table S1). The most stable reference genes after performing the analysis with Bestkeeper (Pfaﬄ et al., 2004) and GeNorm (Vandesompele et al., 2002) were: GAPDH, ETIF3E, and UBI2.

QRT-PCR experiments were run on a QuantStudio 6 Flex Real-Time PCR system (Applied Biosystems-Life Technologies, Carslbad, CA, USA) after samples were aliquoted using a Biomek 3000 Laboratory Automation System (Beckman Coulter, Brea, CA, USA). Reactions were performed in 10 μL with 5 μL of SYBR-green, 2.5 μL of the pair of mixed primers (3.2 μM), and 15 ng of cDNA (2.5 μL of a 1:40 dilution of the synthesized cDNA). Cycling conditions were: 95°C for 2 min followed by 40 cycles of 95°C for 30 s, 60°C for 1 min. A melting curve stage was added: 95°C for 15 s, 60°C for 1 min, and 95°C for 15 s.

Experiments were performed with four biological replicates (one plant = one replicate) for each combination of treatment and time point and three technical replicates for each biological replicate. The geometric mean of the three reference genes selected was used to perform relative quantification of expression using the 2^-ΔΔCT^ method (Livak and Schmittgen, 2001). Statistical analysis to find significant differential expression was performed using *t* tests with an Excel macro.

### CDC Bethune Transcriptome Response

#### Experimental Design

Full transcriptome response and the progression of molecular events were assessed for CDC Bethune plants that were either inoculated with the most aggressive fungal isolate (#81) or with sterile water following the procedures outlined above. Harvesting was performed at 2, 4, 8, and 18 DPI and six biological replicates were collected for each treatment and time point combination. Disease symptoms were scored and plants were harvested and frozen in liquid nitrogen for RNA extractions as aforementioned. RNA samples were pooled in groups of three (to decrease variability), resulting in two pooled biological replicates per treatment and time point, which were used for RNA-seq.

#### RNA-seq

Twenty-seven micrograms of RNA per pooled sample were sent to the Beijing Genomics Institute (BGI) for sequencing. In brief: total RNA was enriched using oligo (dT) magnetic beads, and then fragmented into short fragments (200 bp). The first strand was synthesized using random hexamers, prior to second strand synthesis. The double stranded cDNA was purified using the QiaQuick PCR purification kit (QIAGEN, Venlo, Netherlands), and washed with EB buffer (from the kit) for end repair and addition of base A. Sequencing adapters were ligated to the fragments, before agarose gel electrophoresis purification and enrichment via PCR. The library products were sequenced using and Illumina HiSeq 2000 (Illumina, San Diego, CA, USA) as single-end reads. Raw reads in fastq format were filtered with an in-house pipeline to remove adaptors, remove reads with unknown bases (more than 5%), and remove low quality reads (reads with more than 50% of bases with a quality value equal or less than 10). Reads were deposited in the NCBI sequence read archive as accession PRJNA232613.

Reads from each filtered fastq file were mapped to the flax genome and the flax genome gene models produced previously by our group (Wang et al., 2012a) using TopHat (Trapnell et al., 2009). Mapped reads and the gene models file were used as input for cuﬄinks v2.2.1 to generate transcripts and quantify differential expression. Cuﬄinks was run with the GTF-guide option using the previously annotated gene models file; fragment bias correction and multi-read correction were also applied. The gtf files from transcripts from all treatment and replicates were combined using cuffmerge. Cuffquant was performed using the merged file and cuffdiff was performed comparing the water controls to the inoculated plants in each one of the four time points post-inoculation. The levels of expression were quantified using Fragments Per Kilobase of transcript per Million fragments mapped (FPKM) and significant differential expression was assessed using the Benjamini-Hochberg correction for multiple comparisons (Benjamini and Hochberg, 1995).

To validate differential expression of the genes, qRT-PCR was performed using primers for five mainly upregulated, five mainly downregulated, and five genes with no change in expression, using the same qRT-PCR conditions previously mentioned.

#### *In* silico Analyses

To find gene regulation changes between inoculated and control plants, a systematic process of transcript annotation and differential expression analysis was performed. Since many new unannotated transcripts were found after the RNA-seq analysis, we performed an annotation of close to 50,000 transcripts, which included unannotated and previously annotated flax genes. The merged gtf file from cuffmerge bearing all transcripts was used along the genome fasta file as input for Transdecoder^1^, which uses a Perl script to construct a fasta file of all transcripts. The fasta file was parsed to obtain only the longest isoform from each gene for further annotation.

We annotated all transcripts using 12 cores (physical memory = 2000 mb/core) on a server at Westgrid/Compute Canada^2^. We performed blastx against the non-redundant (nr) GenBank database, the two databases from Uniprot (Trembl and Swissprot) and the TAIR10 protein release. We restricted our search to a maximum of 20 hits with an *e*-value threshold of 10^-10^. The XML output file was loaded into blast2go (Conesa et al., 2005), where the description of the blast hits in each case was compiled as the most common term found in the 20 resulting top hits for each transcript.

The TAIR10 hit IDs from significant differentially expressed genes (*q* < 0.05, after Benjamini-Hochberg multiple testing correction) were used as input for gene ontology (GO) enrichment analysis using AgriGO (Du et al., 2010). The parameters were as follows: species – *A. thaliana*, statistical test – hypergeometric distribution, multi-test adjustment – Yekutieli, significance level – 0.05, minimum number of mapping entries – 5. As background we used a compiled list of all the RNA-seq transcripts that had at least 10 read alignments. We also used plantGSEA (Yi et al., 2013) to find enriched pathways (PlantCyc gene sets and KEGG) using the same parameters as for AgriGO. To see the gene expression changes off all differentially expressed genes at any time point we used multi-experiment viewer MeV4.9 (Saeed et al., 2003).

## Results and Discussion

### Differential Response of Two Flax Cultivars to *F. oxysporum* f. sp. *lini*

**FIGURE 2:**
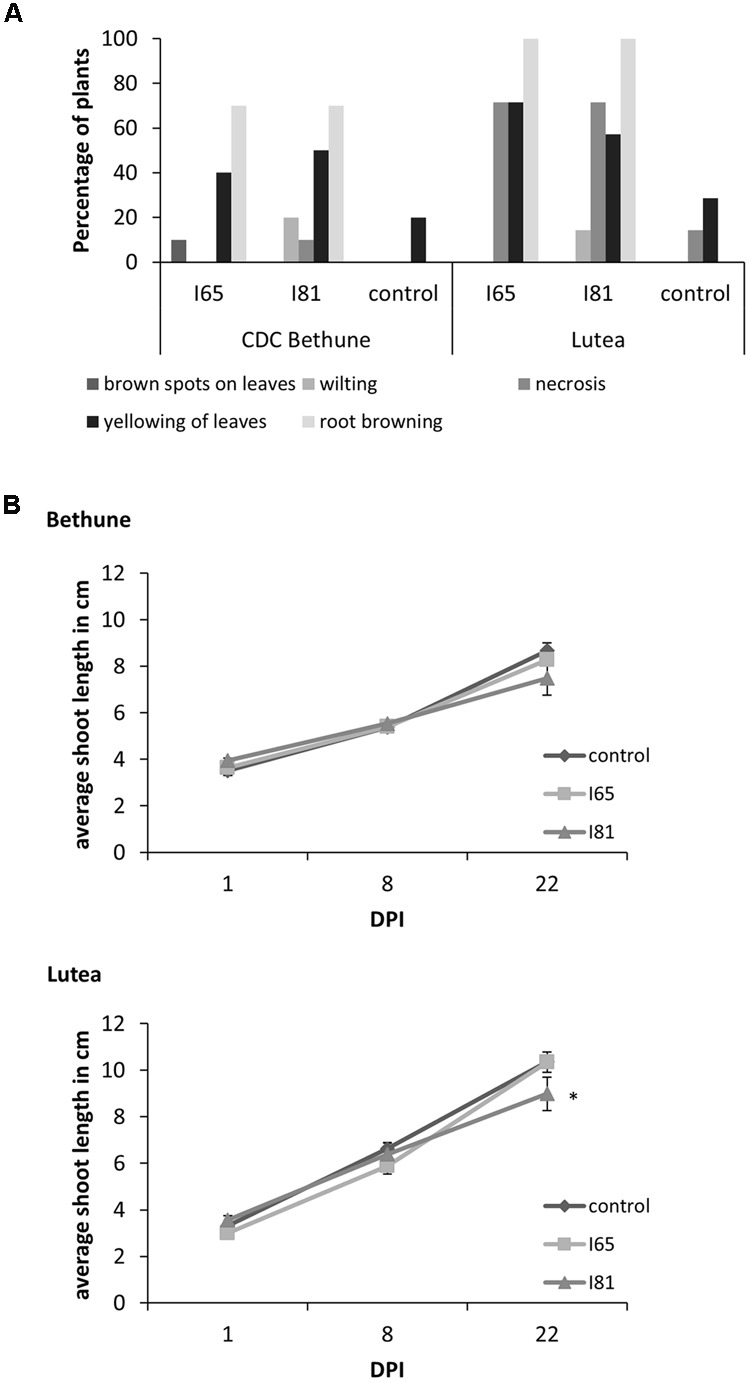
**Disease symptoms and shoot length growth inhibition upon *Foln* infection. (A)** Percentage of plants presenting disease symptoms 22 DPI in the flax cultivars CDC Bethune and Lutea due to the infection with isolates #65 and #81 (indicated as I65 and I81 on the figure) of *Fusarium oxysporum* f. sp. *lini*. **(B)** Difference in average shoot length between control plants and plants inoculated with isolates #65 and #81 for cultivars CDC Bethune and Lutea. An asterisk (^∗^) denotes a significant difference between the control plants and the isolate #81 Lutea plants at day 22 (one tail *t-*test, *p* = 0.04). Error bars = standard error; DPI = days post-inoculation.

**FIGURE 3:**
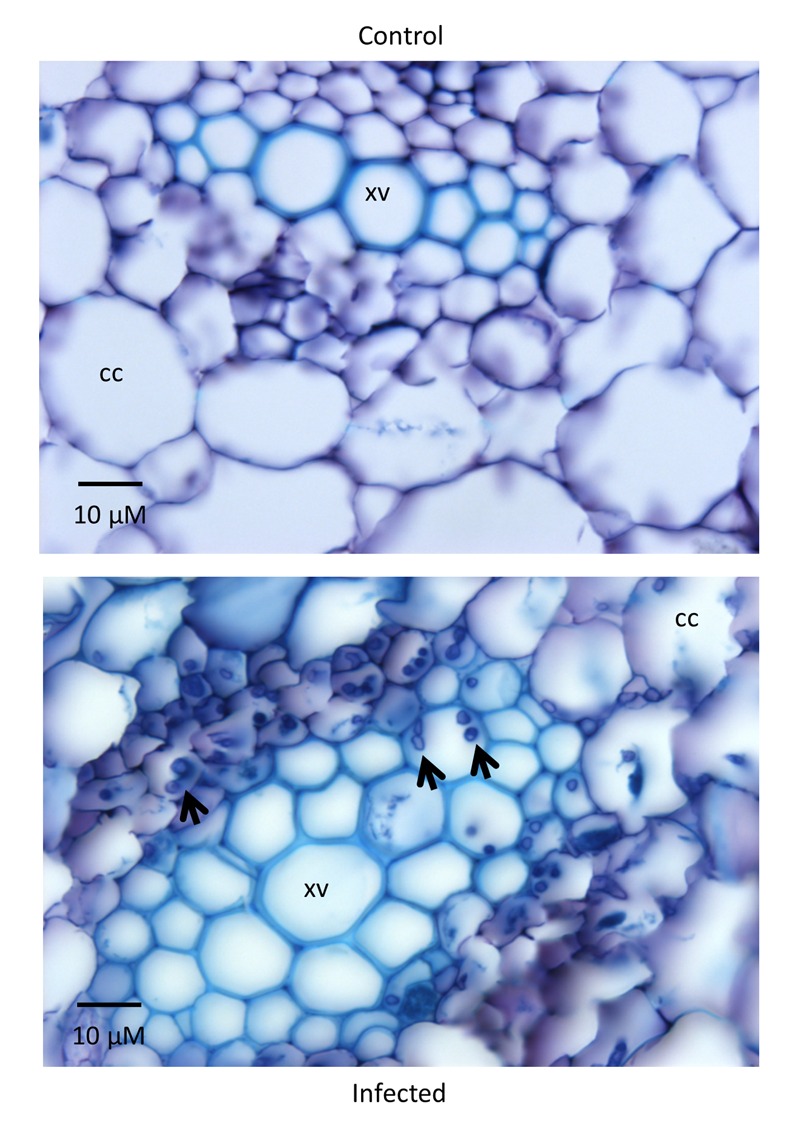
**Root sections of Lutea plants 22 DPI.** The control plant (inoculated with water) on the top shows no signs of infection while the treated plant (isolate #81 inoculum) on the bottom has hyphae (indicated by arrowheads) colonizing the cortical cells (CC) and the xylem vessels (XV). Sections of 12 μm were stained with toluidine blue.

CDC Bethune is an elite, brown-seeded linseed cultivar of flax that is widely grown in Canada and has been reported to have moderate resistance to fusarium wilt (Rowland et al., 2002). To confirm that CDC Bethune was relatively resistant, we conducted preliminary experiments with a panel of linseed varieties selected in consultation with a flax pathologist (Khalid Rashid, *personal communication*), and identified Lutea (a yellow-seeded variety) as a candidate cultivar that could differ in fusarium wilt resistance from CDC Bethune. We inoculated both cultivars with two *F. oxysporum* f. sp. *lini* isolates (#65 and #81) that demonstrated high spore viability/germination (not shown). CDC Bethune plants generally did not show any symptoms until 22 DPI, but wilting was evident in plants inoculated with isolate #81 (**Figure 1**). In Lutea plants, disease symptoms appeared earlier (8 DPI) than in CDC Bethune (22 DPI) and consequently the disease state was more advanced at 22 DPI, with some plants having undergone complete necrosis (**Figure 1F**). Disease symptoms recorded at 22 DPI included yellowing of leaves, brown spots on leaves, wilting, necrosis, and root browning. While most of these characteristics were variable and some infected plants presented little or no symptoms, root browning (represented as a general brown-ashy appearance indicative of rot) was a consistent symptom of disease in both cultivars and with both fungal isolates (**Figure 2A**). When using shoot length to assess the influence of the fungal inoculations on plant growth (Kroes et al., 1998b), there was a significant difference between the shoot lengths of control plants when compared with the lengths of isolate #81 inoculated Lutea plants (**Figure 2B**); nevertheless both cultivars had a 13% shoot length reduction with isolate #81 at 22 DPI. Together, these results showed that isolate #81 was the most aggressive *F. oxysporum* f. sp. *lini* isolate, and that CDC Bethune was more resistant to *F. oxysporum* f. sp. *lini* than Lutea, under our experimental conditions. We were able to re-isolate the fungus from surface-sterilized roots of previously inoculated plants of both CDC Bethune and Lutea (Supplementary Figures S1A,B). Colony and spore morphologies were consistent with the original inocula (Supplementary Figures S1C and S2). As further evidence of infection, we also stained sections of inoculated roots with toluidine blue. Hyphal development in root sections was advanced at 22 DPI, at which point hyphae had colonized the cortical cells and penetrated xylem vessels (**Figure 3**).

### Chitinase Differential Expression

**FIGURE 4:**
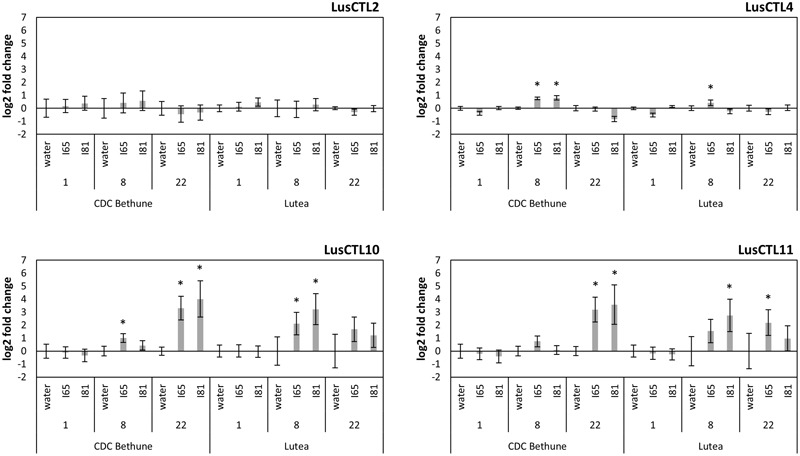
**Log2-fold expression changes through a time course, of four chitinase genes in two flax cultivars (CDC Bethune and Lutea) upon *Foln* inoculation.** Water: control plants, I65: fungal isolate #65 inoculated plants, I81: fungal isolate #81 inoculated plants. Numbers below each treatment indicate days post-inoculation. Error bars are the standard error of the ΔΔCt values (log2-fold changes) calculated as the square root of SEM^2^_(Δcontrol)_ + SEM^2^_(Δtreatment)_. Asterisks denote significant differences between the inoculation treatment and the respective water control (one-tailed *t*-test, *p* < 0.05). Names of chitinases correspond to those referenced in Mokshina et al. (2014).

We next characterized a time course of molecular-scale responses to infection, in both CDC Bethune and Lutea. As markers of the response to fungal infection, we used quantitative PCR to measure transcript abundance of four Glycosyl Hydrolase family 19 (GH19) chitinase genes of flax (Mokshina et al., 2014). These chitinases were selected based on homology to *A. thaliana* genes that had been previously characterized as responsive to pathogens or other related processes (Supplementary Figure S3). Three of the four tested chitinases responded to the fungal inoculation (**Figure 4**). LusCTL4 in CDC Bethune showed a significant increase in transcript abundance at 8 DPI with both *F. oxysporum* f. sp. *lini* isolates, as compared to water controls. This chitinase also showed overexpression at 8 DPI in Lutea with isolate #65. The last two chitinases, LusCTL10 and LusCTL11, were the most responsive and over the time course, appeared to increase in abundance in Lutea earlier that in CDC Bethune: chitinases peaked at 8 DPI for Lutea, while for CDC Bethune, the strongest chitinase responses to both fungal isolates occurred at 22 DPI (**Figure 4**). Since qRT-PCR changes in transcript abundance were marked 8 DPI for Lutea, and not until 22 DPI for CDC Bethune, this suggests constitutive defenses or mechanisms to delay the pathogen interaction in the later variety, as has been seen in other pathosystems (Lawrence et al., 2000). These two chitinases are presumed orthologs, of a class I basic chitinase (At3g12500) which was initially classified as a pathogen induced and defense-related protein (Passarinho and de Vries, 2002), and has shown upregulation in other systems upon pathogen incursion (Ascencio-Ibáñez et al., 2008; Nongbri et al., 2012). Therefore, the increase in the two orthologous chitinases represents a biomarker for *F. oxysporum* f. sp. *lini* infection, and can be effectively linked to the defense response of flax.

### Transcriptome Regulation upon *F. oxysporum* f. sp. *lini* Infection in CDC Bethune

Having demonstrated the relative resistance of CDC Bethune to *F. oxysporum* f. sp. *lini* inoculation, we conducted a RNA-Seq experiment to compare transcriptomes of control and inoculated plants at 2, 4, 8, and 18 DPI, following the same parameters as for our first experiment, but with additional sampling time points which could potentially capture molecular responses at higher temporal resolution. Two biological replicates of three pooled plants each were sequenced at each time point for each treatment (**Table 1**). Reads were mapped to a total of 49,998 transcripts, including published gene models and *de novo* assembled fragments. Over 38,000 transcripts with detectable expression in each time point (transcripts had at least 10 reads aligned to each one of them – **Table 2**), made up a total of 40,042 nr transcripts. We used this set of transcripts for all subsequent analyses. No transcripts showed significant difference in abundance between control and treated plants at 2 DPI (*q* < 0.05), but over 100 transcripts were significantly different on each of days 4 and 8 (**Table 2**), and 1,043 were significant 18 DPI. While at 4 DPI there were a few more genes that decreased rather than increased in abundance, at both 8 and 18 DPI the majority of differentially expressed transcripts increased in abundance. This is in agreement with a pattern seen in wheat infected with *Zymoseptoria tritici* where several defense genes are downregulated earlier and upregulated later in the time course (Rudd et al., 2015). Both *Z. tritici* and *F. oxysporum* are hemibiotrophic fungi and it is possible that this gene expression pattern reflects a transition between the biotrophic stage, where perception takes place, and a second phase when the fungus becomes necrotrophic and the plant activates novel plant defenses.

**Table 1:** RNA-seq statistics.

Treatment	Days post-inoculation	replicate	Total number of reads	Total number of reads after filtering	TopHat mapped reads	Mapped reads %
Water	2	1	23,429,302	22,796,701	20,988,107	92.1
		2	21,010,496	20,459,410	18,891,971	92.3
	4	1	21,619,041	21,071,644	19,641,946	93.2
		2	16,789,921	16,305,931	149,99,487	92.0
	8	1	19,010,668	18,410,625	16,735,093	90.9
		2	19,515,487	19,047,923	17,806,731	93.5
	18	1	21,529,887	20,754,161	18,803,585	90.6
		2	21,542,682	20,901,401	19,238,289	92.0
Foln I81	2	1	23,080,270	22,511,052	20,812,618	92.5
		2	21,937,151	21,071,954	19,193,775	91.1
	4	1	17,545,450	16,998,485	15,443,102	90.8
		2	20,411,895	19,846,352	18,120,142	91.3
	8	1	18,541,028	18,098,407	16,694,252	92.2
		2	20,483,606	19,924,128	18,185,793	91.3
	18	1	19,615,184	18,916,943	17,036,447	90.1
		2	22,211,320	21,636,826	17,065,423	78.9
**Total**			**328,273,388**	**318,751,943**	**289,656,761**	**N/A**
**Average**			**20,517,086.7**	**19,921,996.4**	**18,103,547.6**	**90.9**

**Table 2:** Transcript comparison after gene expression analysis.

Days post-inoculation	Number of transcripts with expression^a^	Number of differentially expressed transcripts (*q* < 0.05)^b^	upregulated (*q* < 0.05)^b^	downregulated (*q* < 0.05)^b^
2	38,768	0	0	0
4	38,302	103	48	55
8	38,407	125	79	46
18	38,616	1043	1008	35

*^a^Transcripts where the minimum number of read alignments (*n* = 10) allowed significance testing between the two conditions. ^b^After correction for multiple testing (Benjamini-Hochberg).*

Validation of RNA-seq results was performed by qRT-PCR of 15 genes (**Table 3**) with different expression patterns over the time course. Log_2_-fold changes showed a correlation of 0.83 between the RNA-seq and the qRT-PCR results for all time points and treatment comparisons.

**Table 3:** Log2-fold change (water vs. inoculum) agreement between RNA-seq and qRT-PCR.

Description		Day 2		Day 4		Day 8		Day 18	
Flax gene ID	Gene	RNA-seq	qRT-PCR	RNA-seq	qRT-PCR	RNA-seq	qRT-PCR	RNA-seq	qRT-PCR
Lus10015351	Nitrate transporter	0.0	0.1	-0.9	-0.6	-1.6	-2.4	-3.5	-2.7
Lus10021936	Basic 7s globulin-like	-1.0	0.4	-1.8	-1.4	-2.1	-3.0	-1.2	-1.4
Lus10016424	Conserved protein similar to AER92600	-2.4	-1.8	-2.9	-2.5	-2.1	-1.9	-0.5	-1.0
Lus10005393	Polyphenol oxidase	1.9	2.9	-0.7	0.1	-1.8	-1.9	1.0	1.6
Lus10039487	Auxin-responsive protein iaa7	-0.1	0.0	0.3	0.2	-0.3	-0.9	-1.3	-2.2
Lus10041830	Chitinase	-0.1	0.4	0.3	0.7	0.3	0.8	3.2	2.0
Lus10004808	Leucoanthocyanidin dioxygenase	0.1	0.9	-0.4	0.1	2.1	2.1	6.7	2.1
Lus10020826	Peroxidase	0.4	0.8	-0.4	0.2	0.1	-0.4	2.6	1.0
Lus10019060	Glycosyl hydrolase family protein with chitinase insertion domain	1.4	2.5	0.1	0.3	-0.5	0.2	4.3	1.5
Lus10008930	Major latex protein	0.3	0.7	-0.3	-0.7	0.5	-0.2	1.7	1.6
Lus10027702	Eukaryotic translation initiation factor 3 subunit c	0.0	0.0	0.0	0.0	0.0	-0.2	-0.2	0.0
Lus10025438	Transcription elongation factor 1	-0.1	0.0	0.1	0.0	0.0	-0.1	0.1	0.2
Lus10015458	DNA-directed RNA polymerase I subunit rpa12	0.0	0.1	0.0	-0.1	0.0	-0.1	0.0	0.1
Lus10005425	Trehalose-6-phosphate synthase	-0.1	-0.2	0.0	0.0	0.0	0.0	0.1	0.2
Lus10038622	Eukaryotic translation initiation factor 3 subunit l-like	0.1	0.1	0.0	0.1	0.0	-0.1	-0.3	-0.1

### Functional Categorization of Differentially Expressed Transcripts

We used complementary approaches to categorize the differentially expressed transcripts that we had identified by RNA-seq: GO enrichment analysis using AgriGO (Du et al., 2010); metabolic pathway enrichment analysis using plantGSEA (Yi et al., 2013); and a heatmap time course using MeV4.9 (Saeed et al., 2003). For Gene Ontology we defined a numerical level of hierarchy based on the acyclic graphs created by AgriGO (Supplementary Figure S4), with more general terms having a lower number (e.g., biological process = 1) and more specific terms having higher numbers (**Table 4**). Our two enrichment analysis in each day of the time course guided a more in-depth analysis on gene groups that were relevant in the plant defense response.

**Table 4:** Biological process gene ontology (GO) categories enriched from significantly different genes.

DPI	GO accession	Term^a^	% sequences query	% sequences background	*p*-value	FDR^b^
4	GO:0045087	Innate immune response (5)	14.29	0.93	7.40E-07	5.30E-05
	GO:0006955	Immune response (3)	14.29	0.99	1.20E-06	5.30E-05
	GO:0002376	Immune system process (2)	14.29	0.99	1.20E-06	5.30E-05
	GO:0006952	Defense response (4)	18.37	2.11	2.60E-06	8.90E-05
	GO:0012501	Programmed cell death (4)	10.20	0.59	1.60E-05	0.00043
	GO:0008219	Cell death (3)	10.20	0.78	6.00E-05	0.0012
	GO:0016265	Death (2)	10.20	0.78	6.00E-05	0.0012
	GO:0051707	Response to other organism (4)	14.29	2.19	0.00018	0.0031
	GO:0009607	Response to biotic stimulus (3)	14.29	2.33	0.00026	0.0039
	GO:0051704	Multi-organism process (3)	14.29	2.77	0.00073	0.0098
	GO:0006950	Response to stress (3)	24.49	8.11	0.0017	0.021
	GO:0050896	Response to stimulus (2)	34.69	14.11	0.0022	0.025
8	GO:0009620	response to fungus (5)	7.32	0.56	8.90E-06	0.0021
	GO:0009607	Response to biotic stimulus (3)	12.20	2.33	4.40E-05	0.0052
	GO:0019748	Secondary metabolic process (3)	9.76	1.60	8.30E-05	0.0066
	GO:0051707	Response to other organism (4)	10.98	2.19	0.00014	0.0084
	GO:0006955	Immune response (3)	7.32	0.99	0.00023	0.0089
	GO:0002376	Immune system process (2)	7.32	0.99	0.00023	0.0089
	GO:0006519	Cellular amino acid and derivative metabolic process (4)	10.98	2.43	0.0003	0.01
	GO:0042398	Cellular amino acid derivative biosynthetic process (6)	6.10	0.83	0.00074	0.016
	GO:0019752	Carboxylic acid metabolic process (6)	12.20	3.39	0.00084	0.016
	GO:0006952	Defense response (4)	9.76	2.11	0.00053	0.016
	GO:0043436	Oxoacid metabolic process (5)	12.20	3.39	0.00084	0.016
	GO:0051704	Multi-organism process (2)	10.98	2.77	0.00076	0.016
	GO:0006082	Organic acid metabolic process (4)	12.20	3.39	0.00086	0.016
	GO:0044283	Small molecule biosynthetic process (4)	10.98	2.86	0.00095	0.016
	GO:0042180	Cellular ketone metabolic process (4)	12.20	3.49	0.0011	0.017
	GO:0045087	Innate immune response (5)	6.10	0.93	0.0012	0.018
	GO:0006629	Lipid metabolic process (4)	10.98	3.15	0.0018	0.025
	GO:0050896	Response to stimulus (2)	29.27	14.11	0.0022	0.029
	GO:0006520	Cellular amino acid metabolic process (7)	7.32	1.57	0.0024	0.03
	GO:0006575	Cellular amino acid derivative metabolic process (5)	6.10	1.10	0.0026	0.03
	GO:0044106	Cellular amine metabolic process (5)	7.32	1.65	0.003	0.034
18	GO:0042398	Cellular amino acid derivative biosynthetic process (6)	4.22	0.83	2.20E-11	2.90E-08
	GO:0050896	Response to stimulus (2)	25.84	14.11	6.80E-11	3.10E-08
	GO:0019748	Secondary metabolic process (3)	5.91	1.60	4.90E-11	3.10E-08
	GO:0009699	Phenylpropanoid biosynthetic process (7)	3.21	0.54	3.20E-10	1.10E-07
	GO:0009607	Response to biotic stimulus (3)	7.09	2.33	3.90E-10	1.10E-07
	GO:0006575	Cellular amino acid derivative metabolic process (5)	4.56	1.10	5.20E-10	1.20E-07
	GO:0051707	Response to other organism (4)	6.76	2.19	6.90E-10	1.40E-07
	GO:0044283	Small molecule biosynthetic process (5)	7.94	2.86	9.40E-10	1.40E-07
	GO:0009611	Response to wounding (4)	3.72	0.77	9.30E-10	1.40E-07
	GO:0006950	Response to stress (3)	16.05	8.11	3.10E-09	4.30E-07
	GO:0009698	Phenylpropanoid metabolic process (6)	3.21	0.66	1.20E-08	1.50E-06
	GO:0051704	Multi-organism process (2)	7.26	2.77	2.30E-08	2.70E-06
	GO:0006952	Defense response (4)	6.08	2.11	2.60E-08	2.70E-06
	GO:0009605	Response to external stimulus (3)	5.07	1.60	4.40E-08	4.30E-06
	GO:0019438	Aromatic compound biosynthetic process (6)	3.72	0.95	5.50E-08	5.00E-06
	GO:0042221	Response to chemical stimulus (3)	13.85	7.14	6.40E-08	5.50E-06
	GO:0009813	Flavonoid biosynthetic process (8)	1.86	0.26	1.70E-07	1.30E-05
	GO:0009620	Response to fungus (5)	2.70	0.56	1.60E-07	1.30E-05
	GO:0006519	Cellular amino acid derivative metabolic process (5)	6.25	2.43	3.20E-07	2.30E-05
	GO:0009812	Flavonoid metabolic process (7)	1.86	0.29	7.80E-07	5.40E-05
	GO:0006725	Cellular aromatic compound metabolic process (5)	4.39	1.54	2.20E-06	0.00014
	GO:0009753	Response to jasmonic acid stimulus (5)	2.70	0.68	3.00E-06	0.00018
	GO:0006979	Response to oxidative stress (4)	3.38	1.18	3.00E-05	0.0018
	GO:0044281	Small molecule metabolic process (3)	11.15	6.49	4.00E-05	0.0023
	GO:0009695	Jasmonic acid biosynthetic process (11)	1.01	0.13	6.50E-05	0.0036
	GO:0031408	Oxylipin biosynthetic process (10)	1.01	0.14	9.40E-05	0.0049
	GO:0032787	Monocarboxylic acid metabolic process (7)	4.05	1.72	0.00013	0.0064
	GO:0009694	Jasmonic acid metabolic process (10)	1.01	0.14	0.00013	0.0065
	GO:0031407	Oxylipin metabolic process (9)	1.01	0.15	0.00018	0.0086
	GO:0009404	Toxin metabolic process (4)	0.84	0.11	0.00026	0.011
	GO:0009407	Toxin catabolic process (5)	0.84	0.11	0.00026	0.011
	GO:0009850	Auxin metabolic process (6)	1.18	0.23	0.00037	0.016
	GO:0006629	Lipid metabolic process (4)	5.91	3.15	0.00043	0.018
	GO:0006576	Cellular biogenic amine metabolic process (6)	1.01	0.19	0.00068	0.028
	GO:0010260	Organ senescence (6)	0.84	0.13	0.00071	0.028
	GO:0010033	Response to organic substance (4)	7.77	4.66	0.00085	0.032

*^a^Numbers in parenthesis correspond to the hierarchy level of gene ontology terms in biological process. ^b^FDR, false discovery rate.*

### Enrichment Analyses

#### Day 2

No transcripts differed significantly in abundance between control and treated plants at day 2 (**Table 2**), therefore, no enriched functional categories were identified.

#### Day 4

At day 4 post-inoculation, 12 GO terms were significantly enriched (**Table 4**); the highest level categories (indicating the more general processes) were immune system process, death, and response to stimulus. The more specific categories pointed toward defense and interaction responses with other organisms. Inspection of the transcripts corresponding to these specific categories included disease resistance proteins and PR thaumatin proteins (Supplementary Table S2). From the plantGSEA analysis only the terpenoid backbone biosynthesis pathway and the metabolism of xenobiotics by cytochrome P450 showed significant enrichment (Supplementary Table S3). Uncategorized transcripts represented by multiple hits included GDSL-like lipase acylhydrolase proteins, laccases, bifunctional inhibitor lipid-transfer proteins (LTPs), and major latex-like protein (MLP) 423 (Supplementary Table S2).

#### Day 8

On day 8 post-inoculation, the number of significantly enriched GO categories increased to 21 (**Table 4**). Higher level categories included multi-organism processes, as well as some categories seen on day 4: immune system process, response to stimulus. Among immune system process transcripts there were PR thaumatin proteins, chitinases, and disease resistant proteins. The category with the highest number of hits (response to stimulus) included all genes from immune system process, plus genes such as peroxidases and WRKY transcription factors (TFs). More specific categories indicated for the first time in this time series a direct interaction with another organism (e.g., response to fungus). Furthermore, GO categories associated with primary and secondary metabolism became enriched; these included metabolism of amino acid derivatives, organic acids, and lipids. The category of secondary metabolic process included cytochrome-related polypeptides and glutathione *s*-transferase (GST) family proteins. The category of lipid metabolic process contained mainly GDSL-like lipase acylhydrolases.

The plantGSEA categorization provided additional information about the metabolic pathways enriched at day 8, particularly pathways for the synthesis of phenylpropanoids and flavonoids (Supplementary Table S3). Specific genes involved in these processes included: peroxidases, terpenoid synthases/cyclases, and 2-oxoglutarate and Fe-dependent oxygenase superfamily proteins (Supplementary Table S2).

Several uncategorized transcripts or gene transcripts with a common annotation but not placed in a specific category also showed distinct patterns of accumulation 8 DPI. These included 2-oxoglutarate and Fe-dependent oxygenase superfamily proteins, UDP-glycosyltransferases (UGTs), laccases, LTPs and MLPs; and genes related to primary carbohydrate metabolism including some family 32 glycosyl hydrolases, sugar transporters, and several cell wall modifying enzymes (e.g., xyloglucan endotransglucosylase, pectinesterase, beta-d-xylosidase 1; Supplementary Table S2).

#### Day 18

The greatest number of enriched categories was found 18 DPI (**Table 4**), and the majority of genes in these categories had increased transcript abundance in the inoculated plants as compared to controls. Enriched categories were associated with: organ senescence; metabolism of auxin, jasmonic acid (JA), aromatics, flavonoids, and toxins; and responses to wounding, oxidative stress, JA, and fungus. With reference to metabolism, transcripts annotated as 2-oxoglutarate and Fe-dependent oxygenases, NAD-binding rossmann-fold proteins and cytochrome-related proteins were classified in the amino acid and aromatic-related processes, with the former genes also related to metabolism of flavonoids, phenylpropanoids, and terpenoids. Two other enriched pathways, lipid and monocarboxylic acid metabolic processes, are a source of fatty acids that can result in downstream synthesis of oxylipin and jasmonate derivatives. The GDSL-like lipase acylhydrolases and alpha beta hydrolases were abundant in the category of lipid metabolic process. The categories of response to stimulus and multi-organism process were comprised of common genes of plant defense responses. Receptors of pathogen signals included leucine-rich repeat (LRR) protein kinases and receptor-like proteins (RLPs), while proteins that have a direct effect on the pathogens comprised chitinases and thaumatins. Inhibitors of pathogen disruptive enzymes were represented by diverse protease inhibitors (PIs). Genes related to the oxidative burst/lignification included peroxidases and laccases, and potential controllers of oxidative stress comprised GSTs, which belonged to both the response to stimulus and secondary metabolic process categories. Multidrug transporters that bind cytotoxic compounds for cell removal were part of the response to stimulus category and included ATP-binding cassette (ABC) transporters, transcripts classified as multidrug and toxic compound extrusion (MATE) eﬄux family proteins, and major facilitator superfamily (MFS) membrane proteins. Transcripts with similarity to aquaporins [major intrinsic proteins (MIPs)], and amino acid transporters were also activated.

TFs were found in multiple GO functional categories, and were one of the most numerous and diverse classes of genes activated 18 DPI (Supplementary Table S2), and included: basic helix-loop-helix (bHLH) DNA-binding proteins, C2H2-type zinc finger proteins, WRKY DNA-binding proteins, MYB domain proteins, NAC domain transcriptional regulators, and winged-helix-DNA-binding TFs.

Protein modification or degradation genes spread among multiple categories were represented by increased abundance transcripts of cysteine and aspartyl proteases, ubiquitin-related proteins, and numerous protein kinases: lectin protein kinases, calcineurin B-like (CBL)-interacting protein kinases (CIPK), LRR protein kinases, and mitogen-activated (MAP) kinases.

The plantGSEA categorization provided further information about the metabolic processes that were enriched at 18 DPI, the most prominent of which were biosynthesis of phenylpropanoids and plant hormones (Supplementary Table S3). Other well represented included biosynthesis of flavonoids, glucosinolates, terpenoids and steroids, stilbenoid, diarylheptanoid and gingerol, tropane, piperidine and pyridine alkaloids, and isoquinoline alkaloids. Biosynthetic pathways for the amino acid precursors of many of these compounds were also enriched, including phenylalanine, tyrosine, cysteine, and methionine, arginine, and proline. Pathways for the synthesis of 13-LOX and 13-HPL, as well as alpha-linolenic acid were enriched as was the downstream JA biosynthesis pathway. Finally, carbohydrate related pathways were also enriched, including glycolysis/gluconeogenesis, and metabolism of galactose, starch and sucrose metabolism. Carbohydrate metabolism and cell wall enzymes were highlighted by the presence of other glycosyl hydrolases (e.g., beta glucosidases), expansins, pectin lyases (polygalacturonases), pectin methylesterase inhibitors (PMEIs), and xyloglucan endotransglucosylases.

Among enzymes that were not categorized by our analysis, UGTs were the most abundant with 19 transcripts, and calcium-binding or dependent proteins totalled 14 hits (Supplementary Table S2). Thirty-one transcripts with increased abundance were annotated as 2-oxoglutarate and Fe-dependent oxygenases, although most of these genes function in diverse pathways. Cytochrome-related proteins, NAD-binding Rossmann-fold proteins and peroxidases were among the transcripts with more hits 18 DPI (Supplementary Table S2).

### Changes in Flax Defense Response

#### Pathogen Elicitor Perception

Thirty-three genes including TIR-NBS-LRR, receptor-like kinases (RLKs), RLPs and lectin protein kinases (LecRK), are part of the plant’s pathogen signal perception and transduction network. Most of these genes were activated 18 DPI, but seven were mainly downregulated throughout the time course (Supplementary Figure S5A). Two of these are disease resistance proteins that appeared at days 4 and 8 in the GO analyses (XLOC_041933 and XLOC_008811), and four of the downregulated transcripts are TIR-NBS-LRRs, which would have been expected to increase their abundance upon pathogen attack. Downregulation of such genes has been found to be controlled by host miRNA in many plant-pathogen interactions, but the repression is usually lifted upon pathogen attack (Li et al., 2011; Zhai et al., 2011; Shivaprasad et al., 2012; Zhu et al., 2013a), which is contrary to our results. However, rust miRNAs that match wheat NBS-LRR transcripts are an example of how pathogens can interfere with plant defenses (Mueth et al., 2015). We also observed a receptor-like cytoplasmic kinase (RLCK; transcripts XLOC_015563 and XLOC_030874) which was induced > twofold (log2 scale) by the treatments. This gene is an ortholog of *Arabidopsis* ATG05940, which encodes a RPMI-induced protein kinase (RIPK), that activates effector triggered immunity (ETI; Liu et al., 2011). Other RLKs that showed increased transcript abundance 18 DPI (Supplementary Figure S5A) could be related to recognition of pathogen proteins or sugars (e.g., chitobiose), or to cell death and immunity, as is common with some SERK1 family members (Gao et al., 2009; Wu et al., 2015).

#### Signal Transduction

G-proteins transduce pathogen detection signals from RLKs (Liu et al., 2013). A few G-protein-related transcript abundances differed significantly only at 18 DPI in our experiment (e.g., XLOC_047108, XLOC_030817, XLOC_38580, XLOC_017620 – Supplementary Table S2). It is possible some of these proteins serve as transducers of fungal response as reported for necrotrophic fungi in *A. thaliana* (Llorente et al., 2005; Trusov et al., 2006, 2009).

Downstream of signal reception, calcium acts as a secondary messenger involved in environmental changes, and its influx from the apoplast and vacuoles into the cytoplasm is a classic response to pathogen infections (Dangl and Jones, 2001; Lecourieux et al., 2006; Vidhyasekaran, 2008). Calcium related genes included binding ef-hand family proteins, calcium transporters, and calcium dependent phosphodiesterases. Calcium dependent genes increased significantly in transcript abundance only 18 DPI (Supplementary Figure S5B). The changes in calcium-related gene response are consistent with physiological measurements of calcium influx upon flax root colonization by *F. oxysporum* (Olivain et al., 2003), and other fungi (Zhuang et al., 2012; Xiao et al., 2013; Alkan et al., 2015; Serrazina et al., 2015).

Multiple modifying enzymes act to complete signal transduction. Twelve transcripts related to protein modification and signaling comprised MAPKs, CIPKs, and phosphatases. All of these proteins showed increased transcript abundance 18 DPI (Supplementary Figures S5B,C). CIPKs have been implicated directly in processes like abscisic acid (ABA) perception and signaling; ABA itself, can act as messenger in response to pathogens (Raghavendra et al., 2010).

Most signaling genes (*R*-genes, calcium, and kinases) as well as TFs (see below), were strongly induced 18 DPI, however, several pathogen responsive genes were already active earlier in the cycle at 4 and 8 DPI (e.g., chitinases). A possible explanation is that some defense components may be constitutively activated and may be reinforced upon initial pathogen detection. For example, the chitin signaling process which can result in activation of several defense genes, depends on chitinases degrading fungal cell wall, oligomer detection by the chitin elicitor binding protein (CEBiP), and signal transduction by a LysM domain-containing receptor-like kinase 1 (LysM RLK1; Wan et al., 2008). Examination of these genes showed that several chitinases (FPKMs > 100) and *CEBiP* and *LysM* RLKs (FPKMs > 10) were constitutively expressed throughout the time course (not shown). This explains how detection of pathogen signals could reinforce pathogen responsive genes.

#### Transcriptional Regulation

Transcription factor families commonly reported to be involved in plant defense include: WRKY, ethylene responsive factors (ERFs), basic-region leucine zipper protein (bZIP), MYB, DNA-binding with one finger protein (DOF), Whirly, MYC, and NAC (Jalali et al., 2006). Seventy-six TFs belonging to 16 different families were regulated during pathogen infection (Supplementary Figures S5D,K; Supplementary Table S2). Most of these showed increased transcript abundance by 18 DPI. We noted three WRKY TFs: XLOC_030137, XLOC_027114, and XLOC_024419 that were consistently less abundant at 2, 4, and 8 DPI, but not at 18 DPI. All three of these were annotated as *WRKY70* (Supplementary Table S2). Overexpression of this gene in *Arabidopsis* positively regulates SA-mediated responses and suppresses JA-mediated defenses (Li et al., 2006). However, this was inconsistent with our observed expression of JA-related genes 18 DPI (see below). Other WRKY genes were also responsive at 18 DPI in our study. For example, transcripts of the orthologs of *WRKY75* (XLOC_020440 and XLOC_014625) which increased transcript abundance in our infected flax plants, also increased in abundance in *Brassica napus* upon challenge with *Sclerotinia sclerotiorum* and *Alternaria brassicae* (Yang et al., 2009). Finally mutants of *AtWRKY3*, which also has a flax ortholog induced by infection in our study (XLOC_010407), have shown increased susceptibility to *B. cinerea* (Lai et al., 2008).

MYBs are a large family of TFs of which only a small number are directly involved in the response to pathogens (Jalali et al., 2006; Ambawat et al., 2013). *MYB113* has been previously reported as induced in *F. oxysporum* inoculations on *Arabidopsis* (Zhu et al., 2013b), and seems critical in the production of anthocyanins which comprise specific stages of phenylpropanoid metabolism (Gonzalez et al., 2008). The presumed ortholog of *MYB113* (XLOC_011383, Supplementary Table S2) was the MYB that showed the highest increase in transcript abundance from TFs in our study at 18 DPI (4.1 log2-fold change). Likewise, XLOC_015378 and XLOC_016754, show close similarity to *Arabidopsis MYB108*, which is necessary through the JA pathway for resistance to *B. cinerea* infection (Mengiste et al., 2003).

#### Hormone Regulation

Besides their role in plant growth and development, plant hormones play a complex role in the signaling response to pathogen attack downstream of the initial plant-pathogen recognition (Pieterse et al., 2012). Hormones like JA and ethylene (ET) control the expression of many PR genes and have feedback loops of regulation to already expressed components. Auxins, particularly indoleacetic acid (IAA), are involved in most plant development processes and are known to also repress the stress hormone salicylic acid (SA; reviewed in Pieterse et al., 2012).

Several genes related to JA biosynthesis increased in transcript abundance starting 4 DPI, but it was only 18 DPI when the majority of these transcripts presented increased abundance (Supplementary Figure S5E). Transcripts directly involved in synthesis of jasmonate included: allene oxide cyclase (*AOC*), allene oxide synthase (*AOS*), 12-oxophytodienoate reductase (*OPR*), and lipoxygenase (*LOX*) family proteins (Supplementary Figure S5E). Transcripts that negatively regulate JA transcriptional activity were also present (jasmonate-zim-domain proteins – JAZ; Chini et al., 2007; Fonseca et al., 2009). We also found three transcripts with increased abundance encoding a transcriptional activator of JA responses (*MYC2*; Supplementary Table S2), which is repressed by JAZ proteins (Chini et al., 2007). Simultaneous activation of both JA biosynthetic genes as their repressors (JAZ domain proteins), has also been reported in *Arabidopsis* (Chung et al., 2008; Kidd et al., 2011). This demonstrates there is a balance between JA production and inactivation, probably to impair excessive levels of JA (Chung et al., 2008; Widemann et al., 2013).

Ethylene, another important hormone in signaling pathways, usually acts synergistically with JA in the defense against necrotrophic pathogens (reviewed in Pieterse et al., 2012). We observed increased transcript abundance of two key ethylene biosynthesis enzymes at 18 DPI, corresponding to two transcripts of *ACS* (1-aminocyclopropane-1-carboxylate synthase) and nine transcripts annotated as 2-oxoglutarate and Fe-dependent oxygenases (Supplementary Table S2), which were similar to 1-aminocyclopropane-1-carboxylate oxidase *ACO* (Supplementary Figure S5F). Transcripts of ethylene response factors *ERF1* (XLOC_005021, XLOC039651) and *ERF14* (XLOC_005023) also increased in abundance. The activation of these biosynthetic genes and of ERFs upon *Fusarium* species inoculation in plant hosts (Li et al., 2013; Xiao et al., 2013) and other fungal pathogens (De Cremer et al., 2013) has been documented in other species. *ERF1* is probably one of the most important markers involved in plant defense against fungal pathogens, and has been linked to *Arabidopsis* resistance to both *F. oxysporum* sp. *conglutinans* and *F. oxysporum* sp. *lycopersici* (Berrocal-Lobo and Molina, 2004). *ERF14* has not only proven to be relevant in the defense against *F. oxysporum*, but also regulates the expression of other ERFs including *ERF1* (Oñate-Sánchez et al., 2006). *ERF14* was the most responsive ethylene-related gene in our study, with a 4.3 log2-fold change at 18 DPI (Supplementary Table S2).

Auxins are involved in most plant development processes and are known to also repress SA (reviewed in Pieterse et al., 2012). Two genes corresponding to an auxin-binding RmlC cupin and an auxin-induced *SAUR* (small auxin-up RNA) were clearly repressed 8 DPI (Supplementary Figure S5G). *SAUR* genes are related to cell expansion (Spartz et al., 2012) demonstrating that this process could be impaired at this time point, which is in agreement with the downregulation of this gene in *F. oxysporum*-infected *A. thaliana* roots (Chen et al., 2014). At 18 DPI two CYP450 family genes (*CYP79B2* and *CYP79B3* – Supplementary Figure S6A) that transform tryptophan to indole-3-acetaldoxime [IAOx, a precursor of indole-3-acetic acid (IAA) and indole glucosinolates], (Glawischnig et al., 2004; González-Lamothe et al., 2009), were present, indicating positive auxin regulation. Likewise, IAA amino acid/amido hydrolases increased in transcript abundance 8 DPI (Supplementary Figure S5G), and became significantly regulated 18 DPI (Supplementary Table S2). The activation of these hydrolases should result in an increasing pool of the hormonally active IAA that is critical for plant germination and growth (Rampey et al., 2004). Favoring growth over defense could result in greater disease progression, but there can also be alternative functions for these enzymes. Auxin conjugate hydrolases genes *IAR3* which corresponds to transcripts XLOC_029902 – XLOC_048550, and *ILL6* which corresponds to transcripts XLOC_010014 – XLOC_005151 (Supplementary Figure S5G), were expressed in *A. thaliana* and in *Brassica rapa* upon challenge by microorganisms (Schuller and Ludwig-Müller, 2006; Truman et al., 2010). *IAR3* and *ILL6* not only control auxin metabolism but are involved in deconjugation of JA-Ile which result in hormone turnover and repression of JA-responsive genes (Widemann et al., 2013; Zhang et al., 2016). This pattern also supports the involvement of these genes in JA regulation (see above).

#### PR Proteins

Pathogenesis-related genes accumulate in plants in response to phytopathogens in the processes of hypersensitive response (HR) and systemic acquired resistance (SAR; Linthorst and Van Loon, 1991; Kitajima and Sato, 1999). Among them, chitinases (PR-3,4,8,11 families; Sels et al., 2008), are a first line of defense to directly disrupt the fungal cell wall, which weakens the pathogen and produces oligomers that become elicitors of additional plant defenses. In total six chitinases from classes I, IV, and V showed increased transcript abundance 18 DPI, while two class IV chitinases were mainly upregulated until day 8 (Supplementary Figure S5H). We observed increased significant transcript abundance of chitinases as early as 4 DPI (Supplementary Table S2), but the maximum number of activated chitinases was reached 18 DPI (Supplementary Figure S5H). Chitinases have been shown to be key players in the response to *F. oxysporum* infection in cabbage (Ahmed et al., 2012), tomato (Amaral et al., 2012), and cavendish banana (Li et al., 2012), to *F. graminearum* in wheat (Kong et al., 2005; Xiao et al., 2013), as well as in other plant-fungal interactions (Kawahara et al., 2012; Windram et al., 2012; Teixeira et al., 2014).

Other PR proteins [thaumatins; lipid transfer proteins (LTPs)]; and PIs were also regulated upon *F. oxysporum* f. sp. *lini* infection in flax; these genes are commonly regulated by plants in pathogenic attacks (Kirubakaran et al., 2008; Wang et al., 2010; Kawahara et al., 2012; Li et al., 2013; Xiao et al., 2013; Lowe et al., 2014; Teixeira et al., 2014; Curto et al., 2015). Thaumatins (PR-5) can cause increased permeabilization of the fungal cell wall inflicting direct damage to fungal hyphae (Vigers, 1991; Vigers et al., 1992). Thaumatins presented a homogeneous transcription pattern from beginning to end, with four out of five having increased transcript abundance and only one being repressed constantly (Supplementary Figure S5I). A second group of genes which is also believed to cause membrane permeability in its action against pathogens are the LTPs (Kader, 1996). While LTPs usually transfer lipids across membranes (reviewed in Sels et al., 2008), their induction may also be related to cutin production stimulated by pathogen attack (Kader, 1996). In our study LTPs showed mixed patterns of increased and decreased abundance during the time course, with one group that was activated early, and then repressed either after day 4 or 8 (four transcripts showed high repression at 8 DPI), and a second group with LTPs with higher increased transcript abundance 18 DPI (Supplementary Figure S5J). Finally, PR proteins classified as PIs (PR-6) control pathogen proteases that work against plant cell wall components or in cell degradation to obtain nutrients for pathogen growth (Habib and Fazili, 2007; Sels et al., 2008). Twenty-one kunitz and serine PIs showed high transcript abundances 18 DPI (Supplementary Figure S5K).

#### Oxidative Burst

Recognition of a pathogen and concomitant signal transduction and changes in the calcium status of the cell are triggers of an oxidative burst. ROS can be involved in HR to produce cell death, in crosslinking with glycoproteins to reinforce the cell wall, or couple with other signaling factors to induce SAR (Torres et al., 2006; Xiao et al., 2013; Swarupa et al., 2014). The first step in ROS production is mediated by NADPH oxidase/RBO (Torres et al., 2006). This gene was represented by two transcripts (XLOC_017620 and XLOC_008027) with increased abundance only at 18 DPI (Supplementary Table S2). Central to the oxidative burst are peroxidases and laccases. Peroxidases can catalyze the production of H_2_O_2_, resulting in activation of cell defenses and programmed cell death (PCD) to stop the spread of the disease, or be used for the synthesis of lignin when H_2_O_2_ and phenolic substrates are available for cell wall strengthening (Apel and Hirt, 2004). While four peroxidases actually decreased in transcript abundance 4 and 8 DPI and maintained non-significant repression at 18 DPI (Supplementary Figure S5L), most peroxidases had increased transcript abundance at 18 DPI. Interestingly, the peroxidases with largest increase in abundance (>3 log2-fold change) in our study (XLOC_08857, XLOC_015715, XLOC_015730, XLOC_027206, and XLOC_028680; Supplementary Table S3) presented very low expression across undisturbed tissues (not shown), making them good candidate markers of the host response to the pathogen. Furthermore, XLOC_015730 and XLOC_027206 whose closest *A. thaliana* ortholog is At5g05340 (known as *AtPrx52*), has been directly implicated in lignin formation under normal development (Fernández-Pérez et al., 2015). This gene increases more than 40-fold 21 dpi when *A. thaliana* is infected with the fungus *Verticillium longisporum* (Floerl et al., 2012), which is in the same range of the non-logarithmic fold changes of our transcripts (16.9 and 10.0, respectively; Supplementary Table S2), making it another good candidate for breeding studies. In the meantime, laccases help in the process of lignification (Vanholme et al., 2010) and are therefore useful in the process of cell wall strengthening during pathogen attack (Malinovsky et al., 2014). From seven laccases, two were activated through 8 DPI while the rest had their activation peak 18 DPI (Supplementary Figure S5M).

To offset potentially negative effects of ROS, scavenging enzymes can help balance the localized HR response (Apel and Hirt, 2004; Edwards and Dixon, 2005; Torres et al., 2006). GSTs had decreased abundance at 2 and 4 DPI, but started increasing 8 DPI and were abundant and significantly upregulated 18 DPI along with other enzymes of the ascorbate-glutathione cycle (Supplementary Figure S5N). This is similar to the situation in Chinese white poplar, where expression of multiple GSTs occurs upon stem blister canker infection (Liao et al., 2014). We also found two transcripts (XLOC_00520 and XLOC_032577) orthologous to *Arabidopsis* GST At2g29420, which has been shown to be regulated by both *B. cinerea* and *Pseudomonas syringae* (Fode et al., 2008).

#### Secondary Metabolism

Numerous functional categories and genes related to secondary metabolism were enriched in the treated samples. These compounds synthesized in response to pathogenic infections, can act directly exerting an antimicrobial effect (pathogen membrane disruption and pathogen protein/enzyme alteration), or indirectly as in the case of cell wall reinforcement (e.g., lignification, callose deposition), or as signaling molecules leading to defense responses, HR or PCD (Pusztahelyi et al., 2015).

Phenylpropanoid metabolism is central to secondary metabolite production of defense-related compounds including monolignols and flavonoids (Ferrer et al., 2008). In a previous study flax plants infected with *F. oxysporum* and *F. culmorum* showed regulation of phenylpropanoid genes and the derived metabolites showed increased abundance (Kostyn et al., 2012). Furthermore, application of fungal elicitors from mycelium, including *F. oxysporum* on flax cell suspensions, results in activation of monolignol gene expression (Hano et al., 2006). The phenylpropanoid genes encountered in our study were mostly activated 18 DPI and comprised transcripts that are key in lignin formation: cinnamic acid 4-hydroxylase (*C4H*), cynnamoyl-CoA reductase (*4CL*), cinnamyl alcohol dehydrogenase (*CAD*), and shikimate quinate hydroxycinnamoyltransferase (*HCT*; Supplementary Figure S5O).

Flavonoids were the most represented group of secondary compounds 18 DPI (Supplementary Figure S5P). Flavonoids have high antioxidant capacity which has been used to create increased resistance to *F. oxysporum* and *F. culmorum* through engineering of transgenic flax plants with a multi-construct including chalcone synthase (*CHS*), chalcone isomerase (*CHI*), and dihydroflavonol reductase (*DFR*) from petunia (Lorenc-Kukuła et al., 2007). One of the three transcripts representing *DFR* (XLOC_021910) showed almost a 3 log2-fold increase, while *CHS* (XLOC_046697) had a 2.4 log2-fold change (Supplementary Table S2). Furthermore, the transgenic plants from the aforementioned study had increased levels of anthocyanins, and we found transcripts of both *DFR* and anthocyanidin synthases which are both implicated in anthocyanin biosynthesis (Supplementary Figure S5P). From seven transcripts matching anthocyanidin synthases, five have log2-fold changes >6 (XLOC_015435, XLOC_002414, XLOC_005793, XLOC_001152, and XLOC_032396), representing some of the largest transcript changes from the study. Additional antioxidant capacity in flavonoids can be achieved by glycosylation, which yields more stable flavonoids. The introduction of UGTs in flax plants resulted in increased resistance against *Fusarium* species through the generation of flavonoid glycosides and increased levels of proanthocyanidin, lignans, phenolic acids, and unsaturated fatty acids (Lorenc-Kukuła et al., 2009). We found 19 UGTs in our study, and 18 of them showed increased transcript abundance 18 DPI (Supplementary Figure S6B).

Isoprenoids or terpenoids are a group of chemicals employed in growth and development but also bearing specialized functions against different forms of stress (Throll, 2015). Several genes related to the metabolism of terpenoids showed increased transcript abundance 18 DPI (Supplementary Figure S5Q). PSY, the key controlling enzyme for carotenoid synthesis (reviewed in Nisar et al., 2015), had a 4.4 log2-fold change increase in our study. This enzyme is induced by diverse stresses including salt, drought and temperature (reviewed in Nisar et al., 2015), but we did not find literature on *in vivo* studies linking it to pathogen response. However, the introduction of a bacterial *PSY* gene under the 35S constitutive promoter in flax demonstrated increased resistance against *F. oxysporum* and *F. culmorum* in flax (Boba et al., 2011). Since carotenoids can act as ROS scavengers too (Stahl and Sies, 2003), the induction of key enzymes involved in their metabolism should be critical during oxidative stress triggered by pathogens.

Finally, other secondary metabolism genes with increased transcript abundance were related to glucosinolate synthesis. The two most induced genes involved in glucosinolate synthesis corresponded to cytochrome P450 family genes *CYP79B2* and *CYP79B3* (also important for auxin precursor synthesis). Additional genes responsible for the synthesis of indole glucosinolates from IAOx (*CYP83B1* and *SUR1*; Bak et al., 2001; Mikkelsen et al., 2004) had increased abundance in our study (XLOC_047586, XLOC_010638, Supplementary Table S2), but two additional enzymes for camalexin synthesis (*CYP71A3* and *CYP71B15*; Schuhegger et al., 2006) did not show regulation. Genes involved in indole glucosinolate and camalexin synthesis were highly induced in *A. thaliana* upon *F. oxysporum* infection (Zhu et al., 2013b). Camalexin also generated membrane permeabilization in *Alternaria brassicicola* (Sellam et al., 2007). The importance of these tryptophan-derived metabolites was highlighted by the study of the double mutant *cyp79b2/cyp79b3* in *A. thaliana*, which resulted in increased susceptibility to the fungal pathogen *Verticillium longisporum* (Iven et al., 2012).

#### Transport

Adjustments in molecule transport are usually made upon pathogen attack, but changes in plant transport mechanisms can also benefit the pathogen. For example, modifications of the relative flow of water and amino acids transport can have beneficial effects for the intruder.

Multidrug transporters constitute a large family of proteins that remove cytotoxic compounds from cells using ATP or a proton pump system (Eckardt, 2001). Thirty-seven transcripts (most with increased abundance 18 DPI – Supplementary Figure S5R) belonging to three multidrug transporter superfamilies comprised MATE eﬄux proteins, MFS proteins, and ABC transporters. The importance of these group of genes is exemplified by plant ABC transporters, a large family of ATP-driven pumps that aids in secondary metabolite transport to deter pathogens, and that can be used for detoxification of harmful compounds (fungal toxins; Yazaki, 2006; Kang et al., 2011; Kosaka et al., 2015).

Major intrinsic proteins or aquaporins, have a main role in water and solute transport and help in the homeostasis during plant stress responses (Afzal et al., 2016). Ten MIPs including six plasma membrane intrinsic proteins (PIPs) and four tonoplast intrinsic proteins (TIPs) were mainly repressed during days 4 and 8, but increased their transcript abundance 18 DPI (Supplementary Figure S5S). It is possible that their regulation is controlled by the pathogen to improve invasion into plant tissues due to greater flow of water through these membrane pores which allows easier haustorial development (Casassola et al., 2015); however, another study showed that these membrane water channels could facilitate the conduction of H_2_O_2_ (Dynowski et al., 2008), and support defense signaling.

Consistent with potential host manipulation by the pathogen, we also found increased transcript abundance for all amino acid transporters/permeases detected, with one exception (Supplementary Figure S5T). In *A. thaliana*, mutants for the amino acid permeases *AAP3* and *AAP6*, presented reduced infestation by nematodes which otherwise benefit from increased amino acid transport to the site of infection (Marella et al., 2013). Three transcripts with similarity to *AAP6* (XLOC_017193, XLOC_026557, and XLOC_027088) were represented in our study with increased abundance 18 DPI (Supplementary Figure S5T).

#### Cell Wall

Extensive cell wall modification at 18 DPI was indicated by a diversity of genes that included expansins, endotransglycosylases, and polygalacturonases (Supplementary Figure S5U). However, a subset of the cell wall related enzymes were consistent with enzyme inhibition (e.g., PMEIs). While some cell wall-related genes indicate defense of the plant against *F. oxysporum* f. sp. *lini* (e.g., hydroxyproline-rich glycoproteins, polygalacturonase inhibiting proteins, PMEI proteins), the large majority of genes with increased transcript abundance 18 DPI suggest a modification that would favor the pathogen (Supplementary Figure S5U). Expansins, endotransglycosylases, glucanases, and genes involved in the pectin metabolism, indicate a potential degradation of cell wall components which would provide sugar nutrients to the fungi and ease colonization by the pathogen (Wojtasik et al., 2011; Bellincampi et al., 2014; Malinovsky et al., 2014).

#### Major Latex Proteins

Major latex proteins comprise a group of genes initially isolated from opium (Nessler et al., 1985). An interesting trend throughout the time course was seen for MLPs. From 14 transcripts matching MLPs, 11 were consistently repressed (Supplementary Figure S5V). Although the function of these proteins has not been completely elucidated, there is evidence of their regulation through hormone signaling (Ruperti et al., 2002; Yang et al., 2015; Wang et al., 2016), and activation in response to pathogens (Schenk et al., 2000; Kiirika et al., 2014; Yang et al., 2015). However, our results were opposite to these studies and we found only one study were 16 major latex proteins were repressed in response to oxidative stress in *Arabidopsis* (Stanley Kim et al., 2004).

#### Other Genes

Many other groups of genes emerged during the time course of infection. Many of these genes are used in diverse metabolic processes and therefore are not easily placed in an exclusive category or group.

Cytochrome family proteins (CYP) P450, were the most numerous group of genes showing regulated transcripts after TFs. These genes are part of diverse processes in cells including primary and secondary metabolism. All but four of the transcripts bearing similarity to CYP genes showed increased transcript abundance 18 DPI (Supplementary Figure S6A). Another group of genes, UGTs, can transfer UDP-glucose to different molecules including hormones and secondary metabolites. The transcripts classified under this category were mainly repressed on day 2, with some genes showing increased transcripts at days 4 and 8, and most genes with higher activation 18 DPI (Supplementary Figure S6B).

Other broad groups of genes are those that can modify or degrade other proteins or related compounds. These included enzymes like subtilases, aspartic and serine proteases and ubiquitin genes related to degradation via the 26S proteasome complex (Supplementary Figures S6C,D). These enzymes had uniformly high transcript abundance 18 DPI.

Finally, two groups of enzymes related to the lipid metabolism (GDSL lipases and alpha beta hydrolases – Supplementary Figures S6E,F) comprise genes with broad substrate specificity, and represent diverse functions. While the alpha beta hydrolases had a higher transcript number 18 DPI, the GDSL lipases had one group with increased transcript abundance from days 2 to 8, but with repression on day 18, and another group that mainly followed the opposite pattern.

#### Bringing It All Together: A Model for the Deployment of Flax Defense against *Fusarium*

**FIGURE 5:**
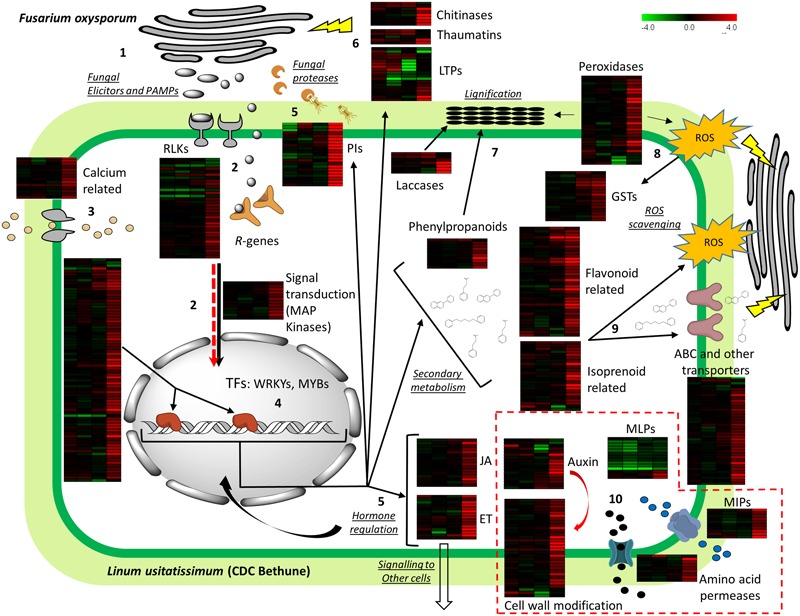
**Model depicting plant defense of flax upon *Foln* inoculation.** Heatmaps for log2-fold gene expression changes at 2, 4, 8, and 18 DPI are shown besides each major gene group analyzed. The full deployment of plant defense is evidenced 18 DPI as seen in the forth column of the heatmaps. (1) During fungal attack, *Fusarium oxysporum* liberates elicitors (pathogen associated molecular patterns – PAMPs-), effectors and fungal proteases (which are also considered effectors) to facilitate infection. (2) Membrane receptors including receptor-like kinases (RLKs), an NBS-LRR (*R-*genes) interact with the PAMPs and effectors, respectively, causing downstream changes in phosphorylation of kinases (e.g., MAP kinases). (3) At the same time an influx of calcium causes changes in calcium-binding proteins that are also involved in signal transduction. (4) Regulation of transcription factors results in activation of hormone-related, defense, and secondary metabolism genes. (5) Presence of jasmonate (JA), ethylene (ET) biosynthetic genes indicates further signaling to other cells and feedback loops to activate more defense genes. (6) Protease inhibitors (PIs) neutralize fungal proteases while chitinases, thaumatins, and lipid transfer proteins (LTPs) act directly on the fungal cell wall or membrane. (7) Lignin precursors are created via phenylpropanoid metabolism and are polymerized into lignin by the action of laccases and peroxidases. (8) Peroxidases are also involved in the generation of reactive oxygen species (ROS) which are regulated by enzymes like glutathione *S*-transferases (GSTs). (9) Flavonoids and isoprenoids can act as antioxidants against ROS, or be directly translocated outside the cell by ATP-binding cassette (ABC) transporters to impair fungal function and growth. (10) Some unexpected regulation was found in some specific transcripts of several gene groups: auxin-related genes, major latex proteins (MLPs), cell wall modification proteins, major intrinsic proteins (MIPs), and amino acid permeases; the potential manipulation of the host by the pathogen to regulate such genes is indicated by a red arrow that parallels signal transduction and by the gene groups surrounded by dashed red lines (see text for explanation).

Based on our observations of the flax transcriptome, we propose a model of the flax defense response to *F. oxysporum* f. sp. *lini* (**Figure 5**). Upon interaction, the fungus potentially liberates PAMPs (pathogen -associated molecular patterns), which are detected by membrane receptors like RLKs (PRRs), triggering innate immune responses via PTI (PAMP-triggered immunity). At the same time, effectors that act as virulence factors are probably deployed and detected by *R*-genes (or the *R-*genes detect changes in the interaction of the effectors with other cell components), in a gene-for-gene resistance fashion (vertical resistance), resulting in ETI (effecter-triggered immunity). A gene with similarity to *RIPK* from *Arabidopsis* was part of this response and may be an important component of resistance (Liu et al., 2011), while four disease resistance proteins downregulated throughout the time course represent interesting targets to study potential immune suppression by the pathogen, probably via small RNAs. The multitude of signal receptor genes indicate that flax (CDC Bethune) uses both non-specific and isolate-specific defenses to deter the pathogen, and therefore can activate both, a general immune response (e.g., PR genes), as well as a HR.

The interactions between PAMPs/effectors and the plant proteins are expected to promote changes in the phosphorylation status of both the interacting plant proteins and downstream proteins of signaling cascades like MAP kinases. Further modifications in calcium-binding proteins also promote the signal transduction process. The result of the signaling processes is a transcriptional reprogramming via numerous TFs that activate hormonal control, PR genes, and secondary metabolism among other processes. From TFs, flax transcripts with similarity to *WRKY3, WRKY70*, and *WRKY75* are responsive in other plant-pathogen interactions (Lai et al., 2008; Yang et al., 2009), and are likely general responders of plant defense against multiple pathogens. On the other hand a transcript with similarity to *MYB113*, which was the most upregulated TF, and is also responsive in the *A. thaliana* – *F. oxysporum* interaction (Zhu et al., 2013b), indicates this could be a good marker gene to inform levels of resistance to *F. oxysporum* infection, if phenotypes can be associated with gene induction.

The regulation of key JA and ET biosynthetic and responsive genes found in this study indicates the importance of these hormones in both the reception and transduction of signals of the defense machinery in and outside the cell. The expression of many PR genes is mediated by these hormones, which also act as signaling compounds in systemic resistance. For example, activation of many defense genes is done via ERFs, from which two regulated transcripts were found in our study (*ERF1* and *ERF14*), both involved in resistance against *F. oxysporum* (Berrocal-Lobo and Molina, 2004; Oñate-Sánchez et al., 2006).

Among PR genes regulated by transcriptional reprograming, chitinases, and thaumatins may directly affect the fungal cell wall and its cell membrane integrity. The presence of LTPs could be related to transport of lipids for cutin deposition, or directly implicated in altering the fungal membrane (Kader, 1996). The transcripts representing these genes showed different expression patterns in the time course with some early expressing genes that were repressed at 18 DPI, and others that were upregulated at this same time point. Additionally, among PR genes were PIs, which had high transcript abundance at 18 DPI, demonstrating that the pathogen has probably entered a necrotrophic phase and has deployed its arsenal to break cell walls.

Polymerization of monolignols generated via the phenylpropanoid metabolism occurs thanks to the presence of laccases and specific peroxidases (flax transcripts had similarity to *AtPrx52*, which is directly implicated in lignin formation; Fernández-Pérez et al., 2015), while some of these peroxidases are also involved in the generation of ROS. The generation of these compounds is highlighted too by the overexpression of transcripts of *NADPH oxidase*. Because we were sampling a mixed population of cells undergoing HR and others getting the signals to deploy defenses but not undergoing cell death, we found indication of regulation of ROS by genes like GSTs and flavonoid biosynthetic genes, but it is likely that ROS is actively involved in damaging both host and pathogen cells at the points of pathogen invasion. Another alternative is that GSTs are hijacked by the pathogen to impair the HR by the plant. Nevertheless, the upregulation of GSTs coincides temporally with peroxidase action showing their importance in ROS regulation.

The other two large groups of regulated secondary metabolism transcripts were flavonoid and isoprenoid-related genes. The products of these metabolic pathways can act as antioxidants but also be translocated (e.g., by ABC transporters) to directly change pathogen cell permeability and interact with membrane proteins, therefore impairing pathogen function (Pusztahelyi et al., 2015). Key enzymes in flavonoid, anthocyanin, and carotenoid production included transcripts of CHS, DFR, anthocyanidin synthases, and phytoene synthase. In fact, this latter group represents some of the transcripts with larger log2-fold changes in our study. Increased expression of some of these enzymes has demonstrated larger resistance against *F. culmorum* and *F. oxysporum* in flax (Lorenc-Kukuła et al., 2007), confirming these genes should be targets to identify natural variation among cultivars, or in gene modification efforts to increase their expression.

Lastly, several gene groups had members with unexpected regulation patterns, which we speculate could be caused by pathogen manipulation. For example, the presence of IAA amido/amino acid hydrolases, as part of auxin genes, could indicate a regulation of JA-conjugates but also results in active IAA which is used in growth. Furthermore, while some cell wall modification genes indicated reinforcement, others like expansins and glucanases weaken the cell wall and could provide an easier entry and nutrients for the pathogen. Under the same hypothesis, amino acid transporters would also provide increased nutrient input, while increased water exchange by aquaporins (MIPs) could facilitate hyphal colonization. Finally, MLPs were unexpectedly downregulated, and although a clear function upon pathogen response has not been established, these genes are usually upregulated in the plant-pathogen interaction.

This is the first transcriptome-wide study of the flax-fusarium interaction, and while confirmatory in many of the expected defense mechanisms of the plant, it also opens new possibilities for the exploration of specific genes. Several genes are candidate markers to explore the disease response across flax cultivars. Additionally, some of the top upregulated transcripts are still unannotated (Supplementary Table S2), which demands further investigation on their function. Both the candidate genes and the highest differentially expressed transcripts should be compared across cultivars to find variability that can be linked to resistance in the phenotypes. If gene variability can be linked to phenotype, the resistance could be engineered back into susceptible cultivars having other desirable production characteristics using gene editing technology. Questions regarding which specific *Avr* genes interact with the plant cell components to suppress immunity, and the cross-kingdom use of small RNAs for transcriptional control, should mark the avenues for new research.

## Author Contributions

LG-G designed and conducted all the experiments, performed all analyses, and wrote the manuscript. MD supervised the research and edited the final version of the manuscript.

## Conflict of Interest Statement

The authors declare that the research was conducted in the absence of any commercial or financial relationships that could be construed as a potential conflict of interest.

## Footnotes

1 https://transdecoder.github.io/

2 https://www.westgrid.ca/--https://www.computecanada.ca/
