# Supplementary material for: RNA-seq Transcriptome Response of Flax (Linum usitatissimum L.) to the Pathogenic Fungus Fusarium oxysporum f. sp. lini
Source: Front Plant Sci. 2016 Nov 24;7:1766. doi: 10.3389/fpls.2016.01766 (PMC5121121; doi:10.3389/fpls.2016.01766)
Supplement: Supplementary file 4 [file Image_1.PDF]

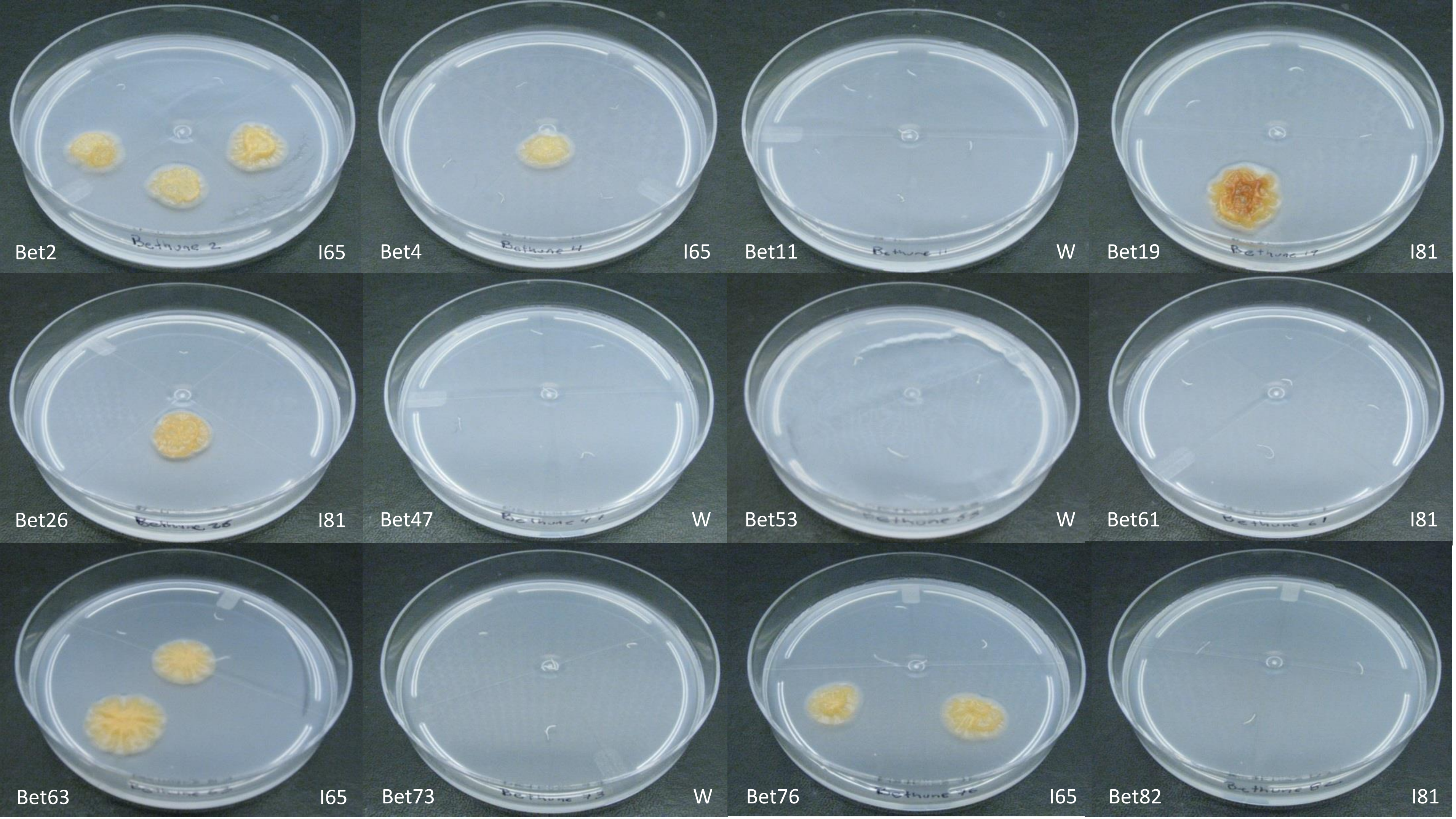
Bet2
I65
Bet4
I65
Bet11
W
Bet19
I81
Bet26
I81
Bet47
W
Bet53
W
Bet61
I81
Bet63
I65
Bet73
W
Bet76
I65
Bet82
I81

(A)

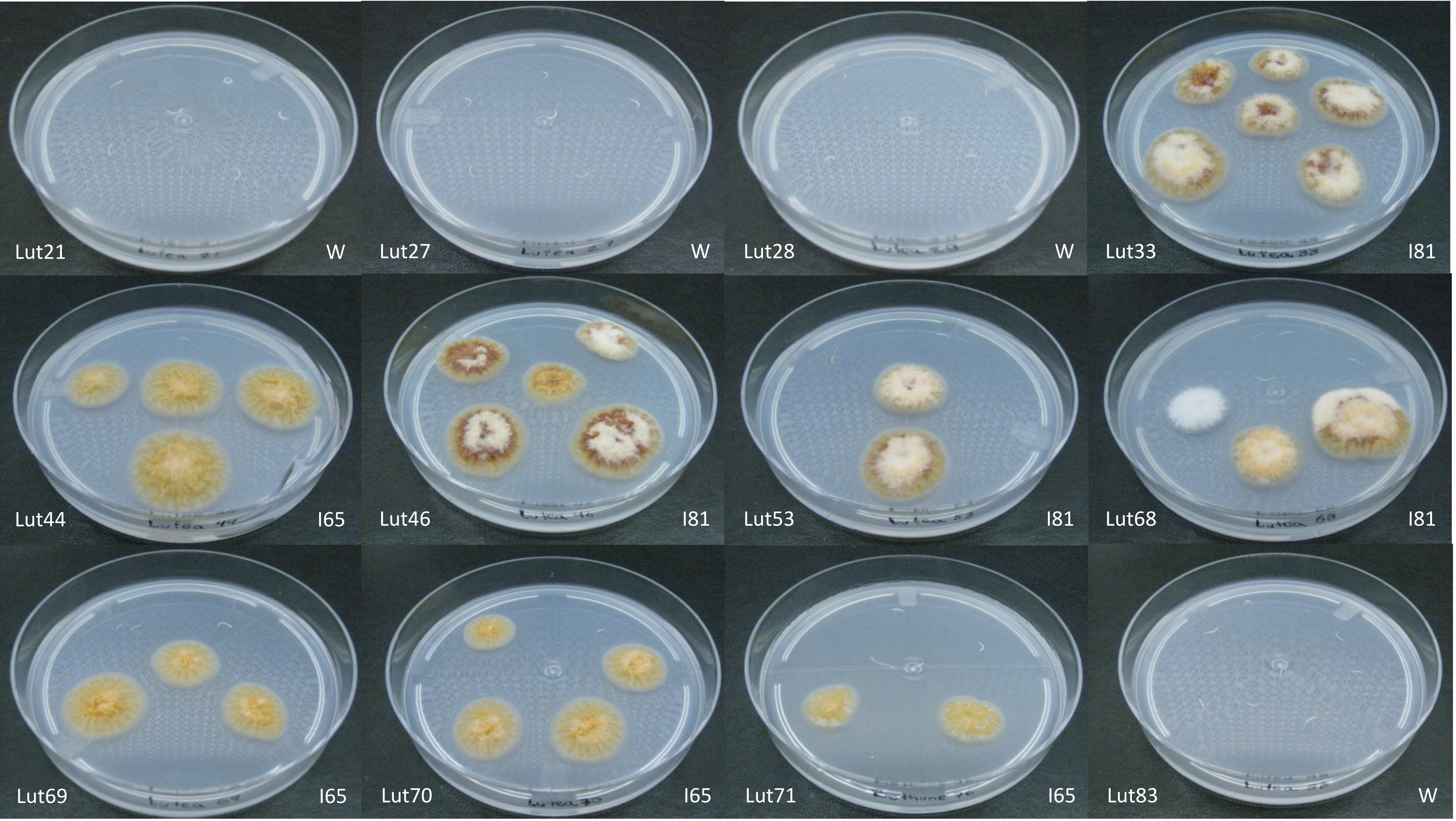
Lut21
W
Lut27
W
Lut28
W
Lut33
I81
Lut44
I65
Lut46
I81
Lut53
I81
Lut68
I81
Lut69
I65
Lut70
I65
Lut71
I65
Lut83
W

(B)

(C)

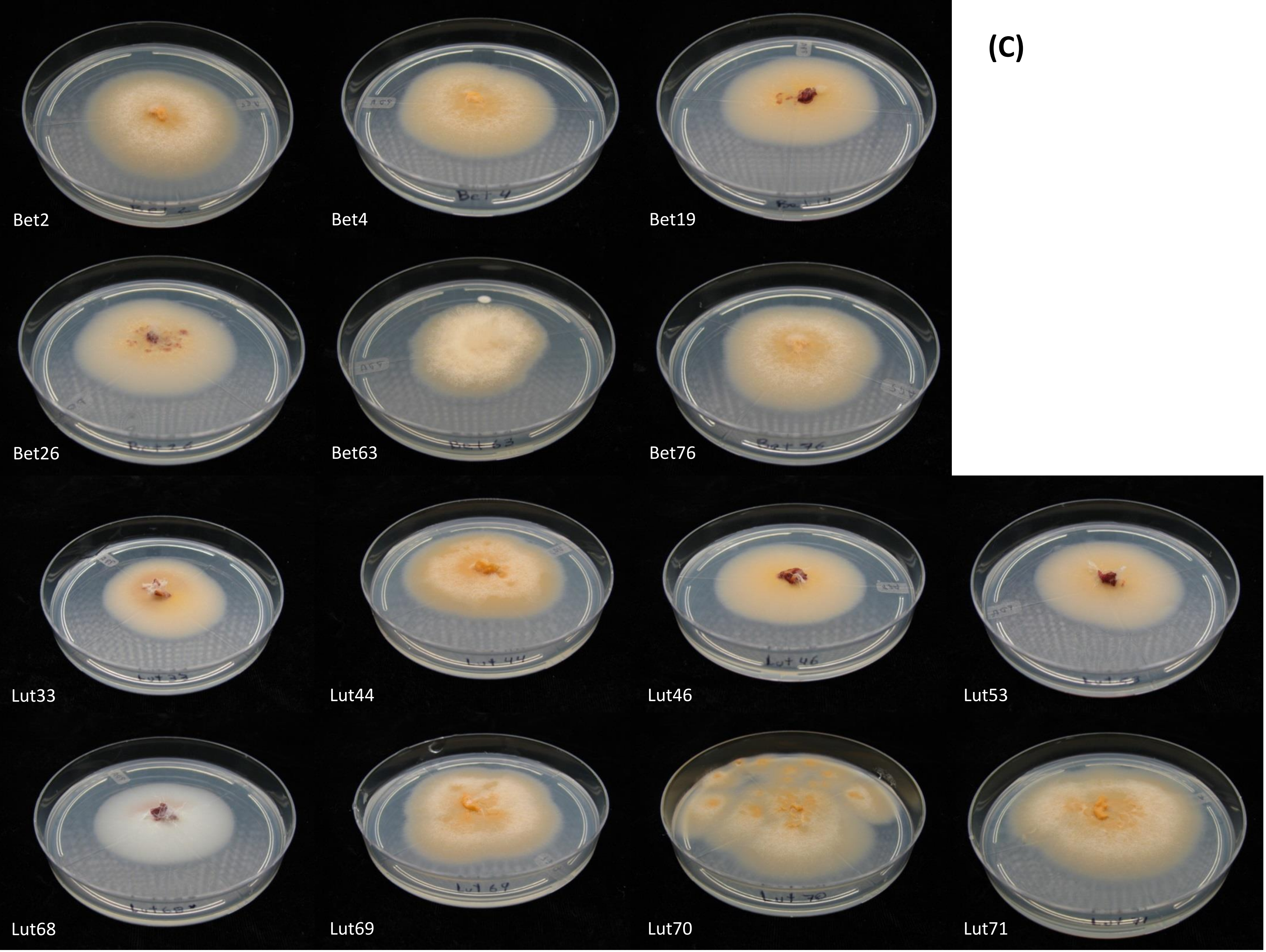
Bet2
Bet4
Bet19
Bet26
Bet63
Bet76
Lut33
Lut44
Lut46
Lut53
Lut68
Lut69
Lut70
Lut71

Figure S1. Reisolation of Foln from surface-sterilized roots. (A) Komada growth for CDC Bethune plants. **(B)** Komada growth for Lutea plants. **(C)** Subculture of fungal isolates from Komada media, grown in PDA. I65 and I81 = fungal isolates, W = water control.
