# Supplementary material for: RNA-seq Transcriptome Response of Flax (Linum usitatissimum L.) to the Pathogenic Fungus Fusarium oxysporum f. sp. lini
Source: Front Plant Sci. 2016 Nov 24;7:1766. doi: 10.3389/fpls.2016.01766 (PMC5121121; doi:10.3389/fpls.2016.01766)
Supplement: Supplementary file 5 [file Image_2.PDF]

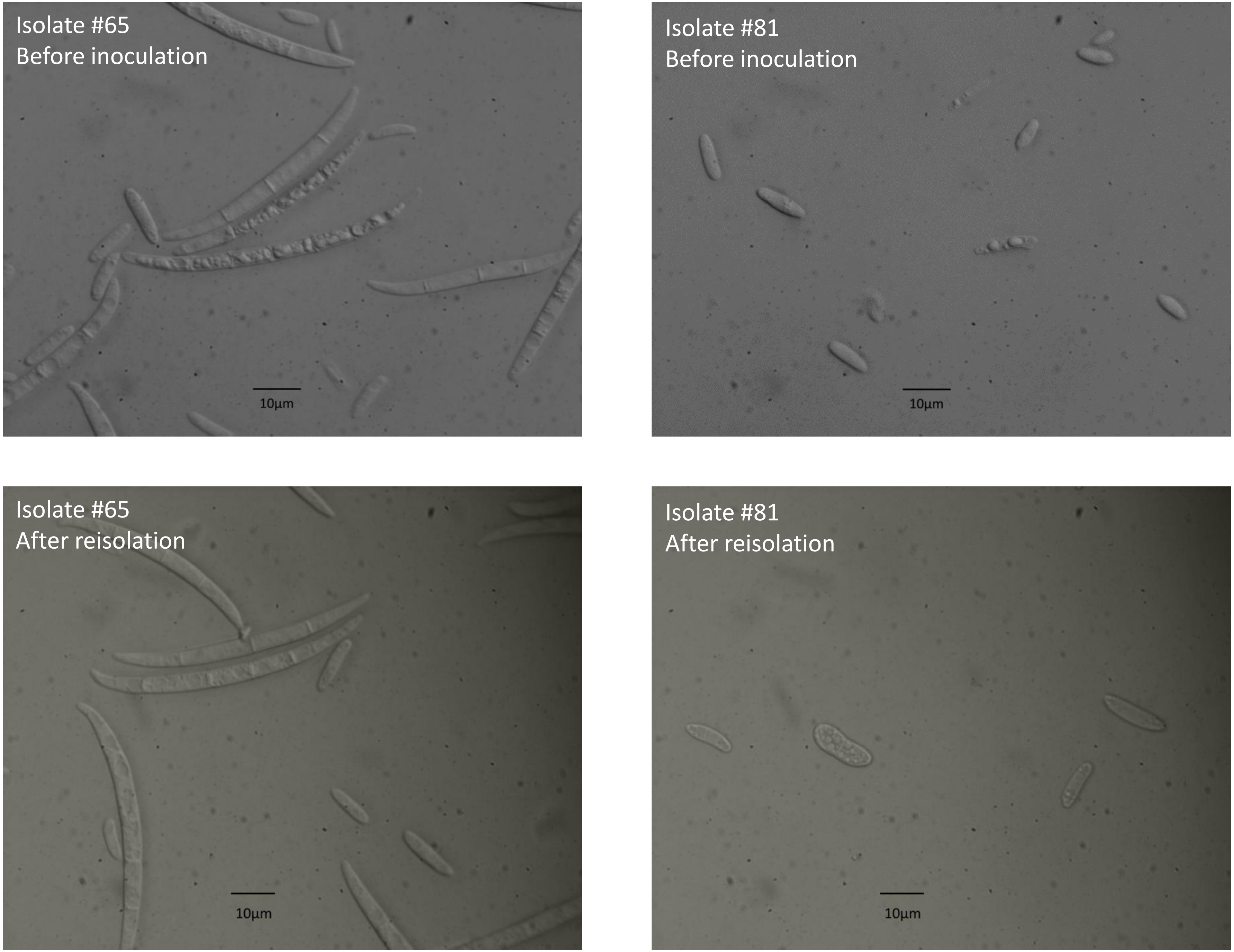
Isolate #65
Before inoculation
10µm
Isolate #81
Before inoculation
10µm
Isolate #65
After reisolation
10µm
Isolate #81
After reisolation
10µm

Figure S2. Comparison of spores pre-inoculation (upper pictures) to spores from PDA subcultures after reisolation from infected roots (lower pictures). While both isolates had macro and microconidia, isolate #65 was dominated by macroconidia while isolate #81 contained more microconidia.
