# Supplementary material for: RNA-seq Transcriptome Response of Flax (Linum usitatissimum L.) to the Pathogenic Fungus Fusarium oxysporum f. sp. lini
Source: Front Plant Sci. 2016 Nov 24;7:1766. doi: 10.3389/fpls.2016.01766 (PMC5121121; doi:10.3389/fpls.2016.01766)
Supplement: Supplementary file 6 [file Image_3.PDF]

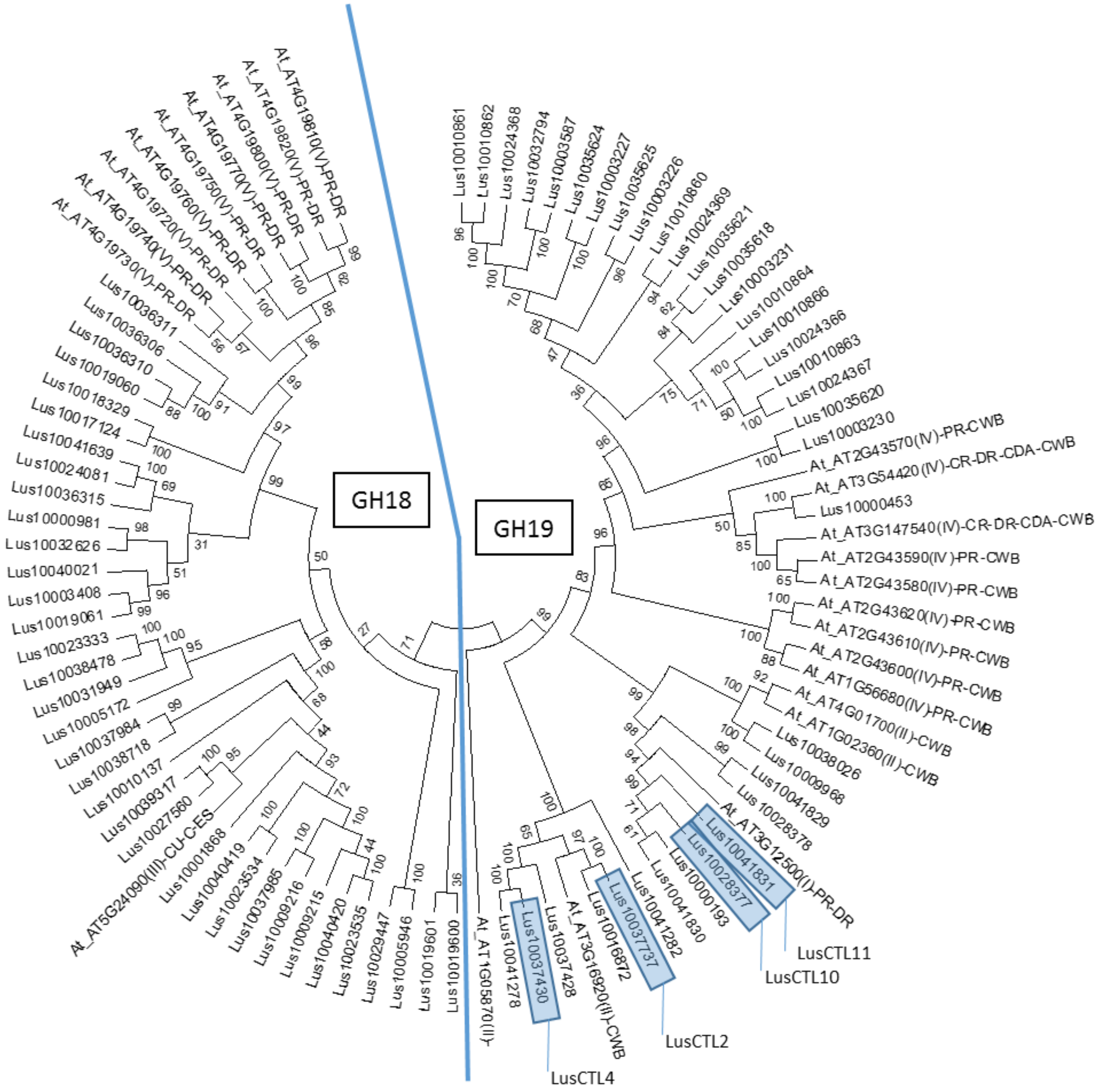
GH18
GH19
At_AT4G19810(V)-PR-DR
At_AT4G19820(V)-PR-DR
At_AT4G19800(V)-PR-DR
At_AT4G19770(V)-PR-DR
At_AT4G19750(V)-PR-DR
At_AT4G19760(V)-PR-DR
At_AT4G19720(V)-PR-DR
At_AT4G19740(V)-PR-DR
At_AT4G19730(V)-PR-DR
Lus10036311
Lus10036306
Lus10036310
Lus10019060
Lus10018329
Lus10017124
Lus10041639
Lus10024081
Lus10036315
Lus10000981
Lus10032626
Lus10040021
Lus10003408
Lus10019061
Lus10023333
Lus10038478
Lus10031949
Lus10005172
Lus10037984
Lus10038718
Lus10010137
Lus10039317
Lus10027560
At_AT5G24090(III)-CU-CES
Lus10001868
Lus10040419
Lus10023534
Lus10037985
Lus10009216
Lus10009215
Lus10040420
Lus10023535
Lus10029447
Lus10005946
Lus10019601
Lus10019600
At_AT1G05870(II)-
Lus10010861
Lus10010862
Lus10024368
Lus10032794
Lus10003587
Lus10035624
Lus10003227
Lus10035625
Lus10003226
Lus10010860
Lus10024369
Lus10035621
Lus10035618
Lus10003231
Lus10010864
Lus10010866
Lus10024366
Lus10010863
Lus10024367
Lus10035620
Lus10003230
At_AT2G43570(IV)-PR-CWB
At_AT3G54420(IV)-CR-DR-CDA-CWB
Lus10000453
At_AT3G147540(IV)-CR-DR-CDA-CWB
At_AT2G43590(IV)-PR-CWB
At_AT2G43580(IV)-PR-CWB
At_AT2G43620(IV)-PR-CWB
At_AT2G43610(IV)-PR-CWB
At_AT2G43600(IV)-PR-CWB
At_AT1G56680(IV)-PR-CWB
At_AT4G01700(II)-CWB
At_AT1G02360(II)-CWB
Lus10038026
Lus10009968
Lus10041829
Lus10028378
At_AT3G12500(I)-PR-DR
Lus10041831
Lus10028377
Lus10000193
Lus10041830
Lus10041282
Lus10037737
Lus10016872
At_AT3G16920(II)-CWB
Lus10037428
Lus10037430
Lus10041278
LusCTL11
LusCTL10
LusCTL2
LusCTL4

Figure S3. Relationship of flax chitinases with previously characterized Arabidopsis chitinases. The tree was done using a muscle alignment (default parameters) and the dendrogram was built under the following parameters: neighbor joining (NJ), 1000 bootstrap replicates (each branch shows % support), p-distance and pairwise deletion. Predicted function was taken from: *Arabidopsis thaliana*: a Genomic Survey, Passarinho and Vries (2002): C – cytokinesis, CDA – cell death and aging, CR – cell rescue, CU – carbohydrate utilization, CWB – cell wall biogenesis, DR – defense related, ES – extracellular secretion, PR – pathogen response. Selected chitinases are outlined in rectangles and their respective labels (e.g. LuCTL14), correspond to a previous report (Mokshina *et al.*, 2014). Chitinase classes are in parentheses. GH = glycosyl hydrolase family.
