# Supplementary material for: RNA-seq Transcriptome Response of Flax (Linum usitatissimum L.) to the Pathogenic Fungus Fusarium oxysporum f. sp. lini
Source: Front Plant Sci. 2016 Nov 24;7:1766. doi: 10.3389/fpls.2016.01766 (PMC5121121; doi:10.3389/fpls.2016.01766)
Supplement: Supplementary file 7 [file Image_4.PDF]

(A)

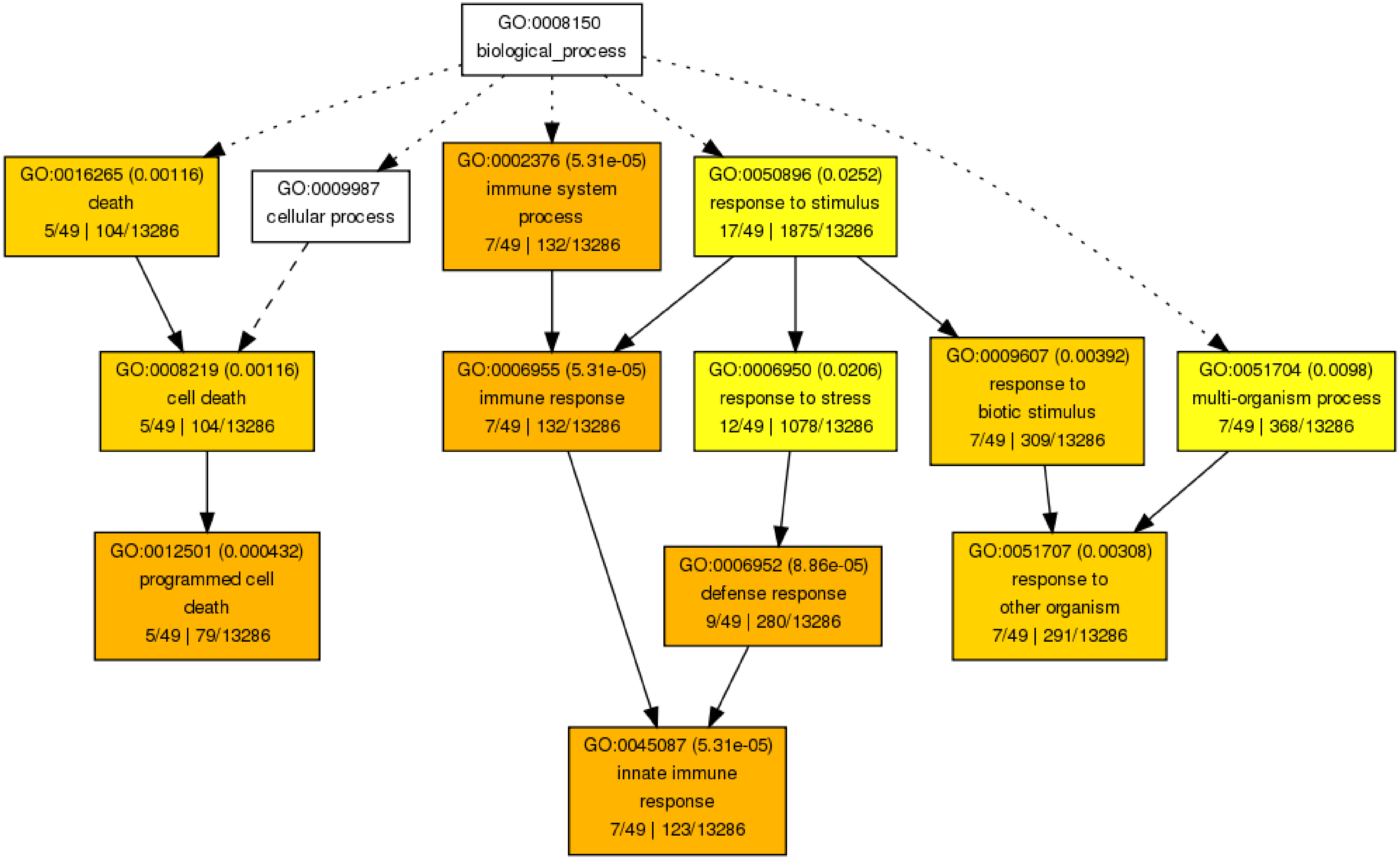
GO:0008150
biological_process
GO:0016265 (0.00116)
death
5/49 | 104/13286
GO:0009987
cellular process
GO:0002376 (5.31e-05)
immune system
process
7/49 | 132/13286
GO:0050896 (0.0252)
response to stimulus
17/49 | 1875/13286
GO:0008219 (0.00116)
cell death
5/49 | 104/13286
GO:0006955 (5.31e-05)
immune response
7/49 | 132/13286
GO:0006950 (0.0206)
response to stress
12/49 | 1078/13286
GO:0009607 (0.00392)
response to
biotic stimulus
7/49 | 309/13286
GO:0051704 (0.0098)
multi-organism process
7/49 | 368/13286
GO:0012501 (0.000432)
programmed cell
death
5/49 | 79/13286
GO:0006952 (8.86e-05)
defense response
9/49 | 280/13286
GO:0051707 (0.00308)
response to
other organism
7/49 | 291/13286
GO:0045087 (5.31e-05)
innate immune
response
7/49 | 123/13286

(B)

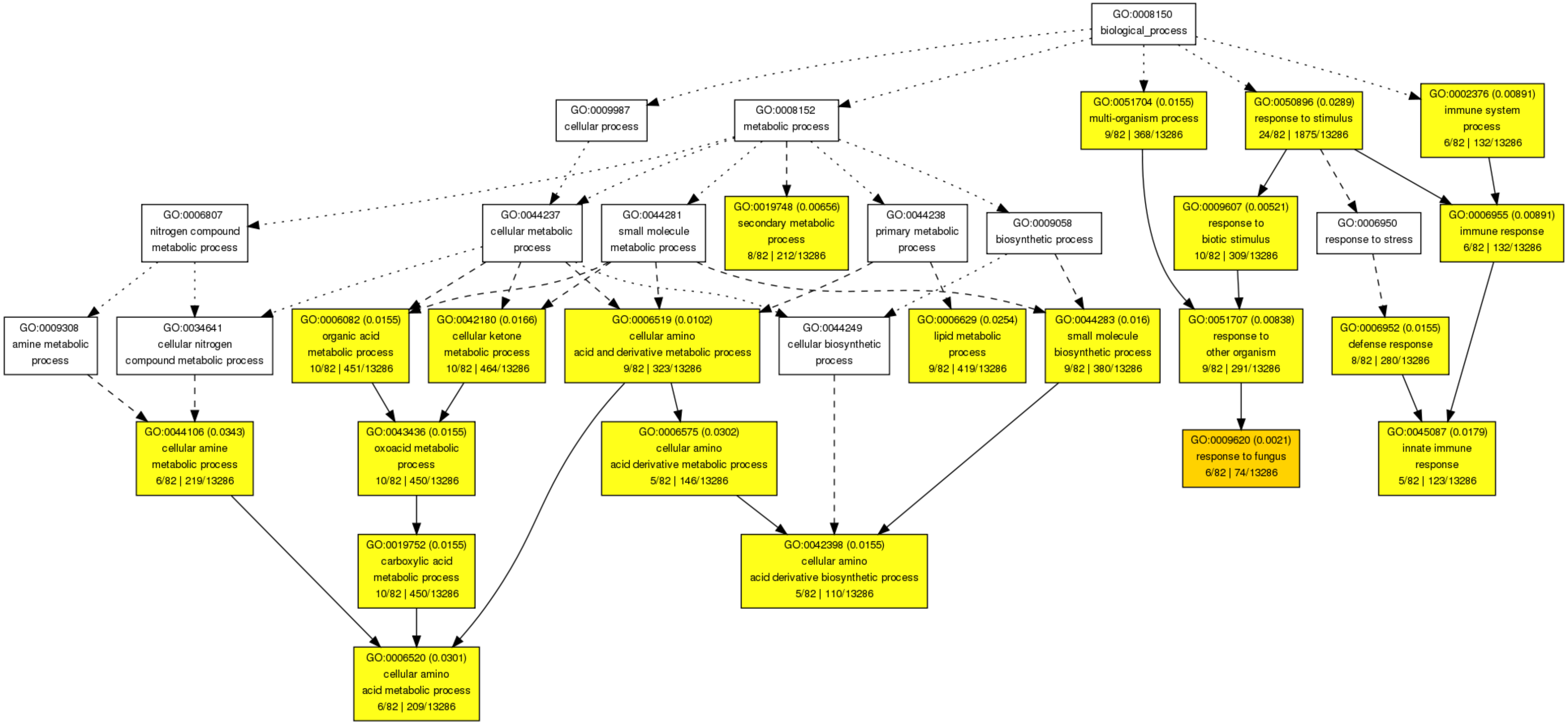
GO:0008150
biological_process
GO:0009987
cellular process
GO:0008152
metabolic process
GO:0051704 (0.0155)
multi-organism process
9/82 | 368/13286
GO:0050896 (0.0289)
response to stimulus
24/82 | 1875/13286
GO:0002376 (0.00891)
immune system process
6/82 | 132/13286
GO:0006807
nitrogen compound metabolic process
GO:0044237
cellular metabolic process
GO:0044281
small molecule metabolic process
GO:0019748 (0.00656)
secondary metabolic process
8/82 | 212/13286
GO:0044238
primary metabolic process
GO:0009058
biosynthetic process
GO:0009607 (0.00521)
response to biotic stimulus
10/82 | 309/13286
GO:0006950
response to stress
GO:0006955 (0.00891)
immune response
6/82 | 132/13286
GO:0009308
amine metabolic process
GO:0034641
cellular nitrogen compound metabolic process
GO:0006082 (0.0155)
organic acid metabolic process
10/82 | 451/13286
GO:0042180 (0.0166)
cellular ketone metabolic process
10/82 | 464/13286
GO:0006519 (0.0102)
cellular amino acid and derivative metabolic process
9/82 | 323/13286
GO:0044249
cellular biosynthetic process
GO:0006629 (0.0254)
lipid metabolic process
9/82 | 419/13286
GO:0044283 (0.016)
small molecule biosynthetic process
9/82 | 380/13286
GO:0051707 (0.00838)
response to other organism
9/82 | 291/13286
GO:0006952 (0.0155)
defense response
8/82 | 280/13286
GO:0044106 (0.0343)
cellular amine metabolic process
6/82 | 219/13286
GO:0043436 (0.0155)
oxoacid metabolic process
10/82 | 450/13286
GO:0006575 (0.0302)
cellular amino acid derivative metabolic process
5/82 | 146/13286
GO:0009620 (0.0021)
response to fungus
6/82 | 74/13286
GO:0045087 (0.0179)
innate immune response
5/82 | 123/13286
GO:0019752 (0.0155)
carboxylic acid metabolic process
10/82 | 450/13286
GO:0042398 (0.0155)
cellular amino acid derivative biosynthetic process
5/82 | 110/13286
GO:0006520 (0.0301)
cellular amino acid metabolic process
6/82 | 209/13286

(C)

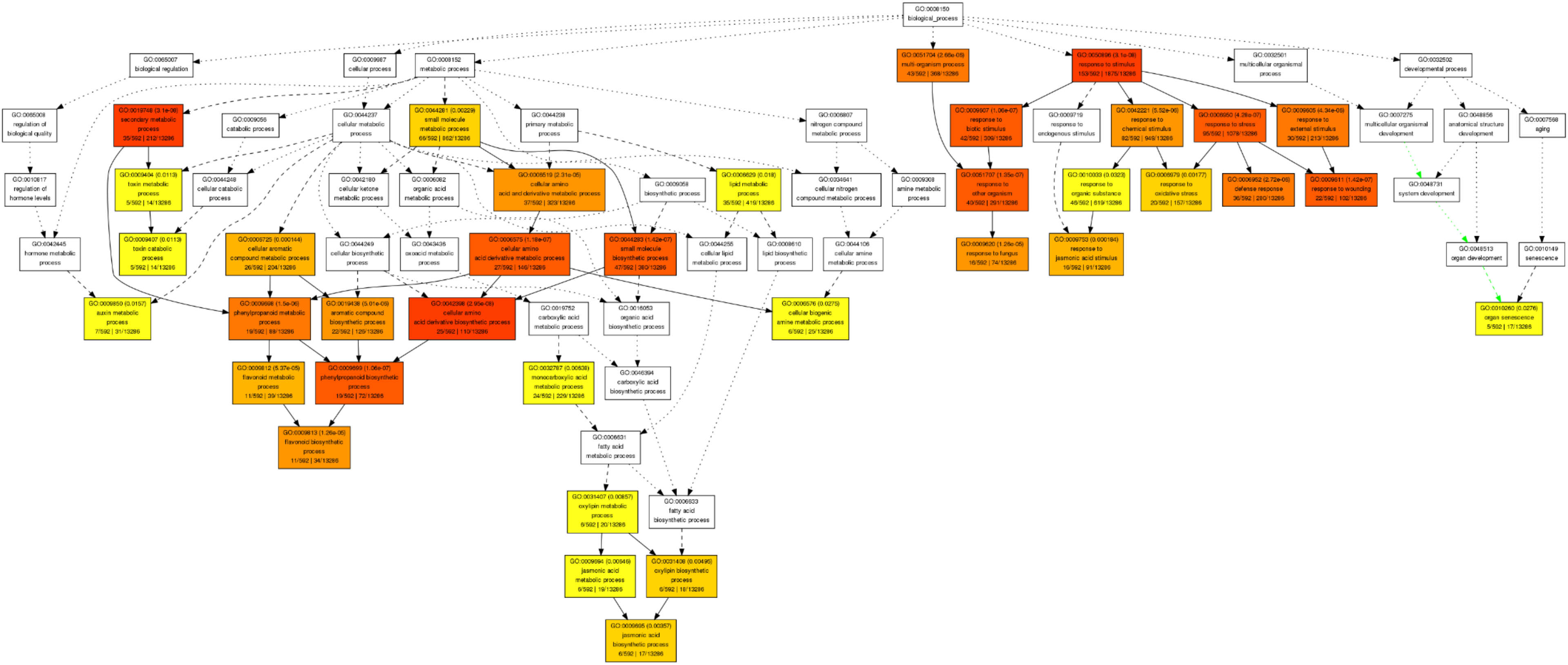
GO:0008150 biological_process
GO:0065007 biological regulation
GO:0009987 cellular process
GO:0008152 metabolic process
GO:0051704 (2.66e-06) multi-organism process 43/592 | 368/13286
GO:0050896 (3.1e-08) response to stimulus 153/592 | 1875/13286
GO:0032501 multicellular organismal process
GO:0032502 developmental process
GO:0065008 regulation of biological quality
GO:0019748 (3.1e-08) secondary metabolic process 35/592 | 212/13286
GO:0009056 catabolic process
GO:0044237 cellular metabolic process
GO:0044281 (0.00229) small molecule metabolic process 66/592 | 862/13286
GO:0044238 primary metabolic process
GO:0006807 nitrogen compound metabolic process
GO:0009607 (1.06e-07) response to biotic stimulus 42/592 | 309/13286
GO:0009719 response to endogenous stimulus
GO:0042221 (5.52e-06) response to chemical stimulus 82/592 | 949/13286
GO:0006950 (4.28e-07) response to stress 95/592 | 1078/13286
GO:0009605 (4.34e-06) response to external stimulus 30/592 | 213/13286
GO:0007275 multicellular organismal development
GO:0048856 anatomical structure development
GO:0007568 aging
GO:0010817 regulation of hormone levels
GO:0009404 (0.0113) toxin metabolic process 5/592 | 14/13286
GO:0044248 cellular catabolic process
GO:0042180 cellular ketone metabolic process
GO:0006082 organic acid metabolic process
GO:0006519 (2.31e-05) cellular amino acid and derivative metabolic process 37/592 | 323/13286
GO:0009058 biosynthetic process
GO:0006629 (0.018) lipid metabolic process 35/592 | 419/13286
GO:0034641 cellular nitrogen compound metabolic process
GO:0009308 amine metabolic process
GO:0051707 (1.35e-07) response to other organism 40/592 | 291/13286
GO:0010033 (0.0323) response to organic substance 46/592 | 619/13286
GO:0006979 (0.00177) response to oxidative stress 20/592 | 157/13286
GO:0006952 (2.72e-06) defense response 36/592 | 280/13286
GO:0009611 (1.42e-07) response to wounding 22/592 | 102/13286
GO:0048731 system development
GO:0042445 hormone metabolic process
GO:0009407 (0.0113) toxin catabolic process 5/592 | 14/13286
GO:0006725 (0.000144) cellular aromatic compound metabolic process 26/592 | 204/13286
GO:0044249 cellular biosynthetic process
GO:0043436 oxoacid metabolic process
GO:0006575 (1.18e-07) cellular amino acid derivative metabolic process 27/592 | 146/13286
GO:0044283 (1.42e-07) small molecule biosynthetic process 47/592 | 380/13286
GO:0044255 cellular lipid metabolic process
GO:0008610 lipid biosynthetic process
GO:0044106 cellular amine metabolic process
GO:0009620 (1.26e-05) response to fungus 16/592 | 74/13286
GO:0009753 (0.000184) response to jasmonic acid stimulus 16/592 | 91/13286
GO:0048513 organ development
GO:0010149 senescence
GO:0009850 (0.0157) auxin metabolic process 7/592 | 31/13286
GO:0009698 (1.5e-06) phenylpropanoid metabolic process 19/592 | 88/13286
GO:0019438 (5.01e-06) aromatic compound biosynthetic process 22/592 | 126/13286
GO:0042398 (2.95e-08) cellular amino acid derivative biosynthetic process 25/592 | 110/13286
GO:0019752 carboxylic acid metabolic process
GO:0016053 organic acid biosynthetic process
GO:0006576 (0.0275) cellular biogenic amine metabolic process 6/592 | 25/13286
GO:0010260 (0.0276) organ senescence 5/592 | 17/13286
GO:0009812 (5.37e-05) flavonoid metabolic process 11/592 | 39/13286
GO:0009699 (1.06e-07) phenylpropanoid biosynthetic process 19/592 | 72/13286
GO:0032787 (0.00638) monocarboxylic acid metabolic process 24/592 | 229/13286
GO:0046394 carboxylic acid biosynthetic process
GO:0009813 (1.26e-05) flavonoid biosynthetic process 11/592 | 34/13286
GO:0006631 fatty acid metabolic process
GO:0031407 (0.00857) oxylipin metabolic process 6/592 | 20/13286
GO:0006633 fatty acid biosynthetic process
GO:0009694 (0.00646) jasmonic acid metabolic process 6/592 | 19/13286
GO:0031408 (0.00495) oxylipin biosynthetic process 6/592 | 18/13286
GO:0009695 (0.00357) jasmonic acid biosynthetic process 6/592 | 17/13286

Figure S4. Gene ontology acyclic graph of enriched biological process terms in days 4 (A), 8 (B) and 18 (C) post-inoculation. The enriched terms are based on differentially expressed genes ($q < 0.05$) when compared to the background population of all genes with at least 10 read alignments from the RNA-seq analysis.
