# Supplementary material for: RNA-seq Transcriptome Response of Flax (Linum usitatissimum L.) to the Pathogenic Fungus Fusarium oxysporum f. sp. lini
Source: Front Plant Sci. 2016 Nov 24;7:1766. doi: 10.3389/fpls.2016.01766 (PMC5121121; doi:10.3389/fpls.2016.01766)
Supplement: Supplementary file 8 [file Image_5.PDF]

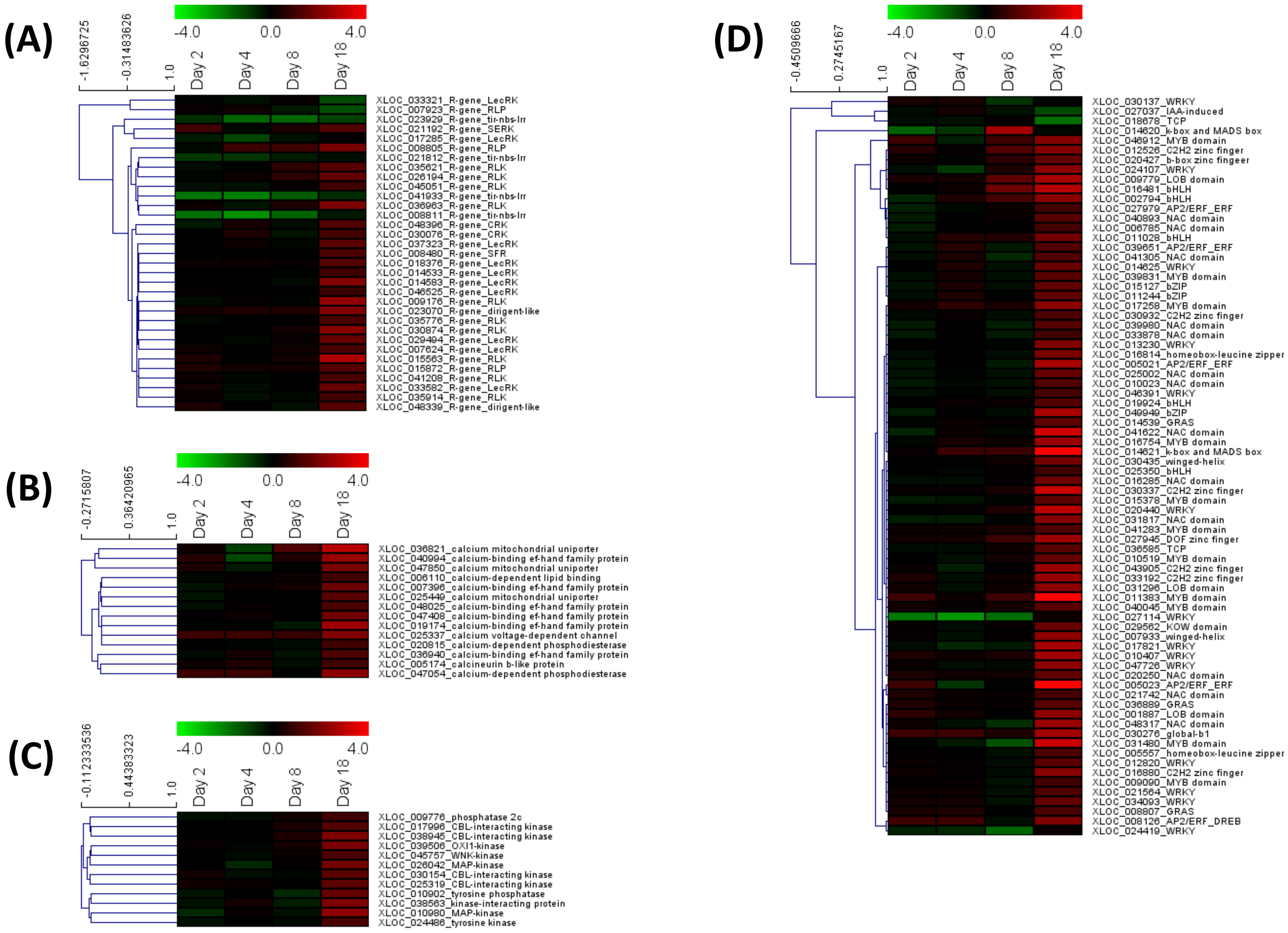
(A)
-1.6296725
-0.31483626
1.0
-4.0
0.0
4.0
Day 2
Day 4
Day 8
Day 18
XLOC_033321_R-gene_LecRK
XLOC_007923_R-gene_RLP
XLOC_023929_R-gene_tir-nbs-lrr
XLOC_021192_R-gene_SERK
XLOC_017285_R-gene_LecRK
XLOC_008805_R-gene_RLP
XLOC_021812_R-gene_tir-nbs-lrr
XLOC_035621_R-gene_RLK
XLOC_026194_R-gene_RLK
XLOC_045051_R-gene_RLK
XLOC_041933_R-gene_tir-nbs-lrr
XLOC_036963_R-gene_RLK
XLOC_008811_R-gene_tir-nbs-lrr
XLOC_048396_R-gene_CRK
XLOC_030076_R-gene_CRK
XLOC_037323_R-gene_LecRK
XLOC_008480_R-gene_SFR
XLOC_018376_R-gene_LecRK
XLOC_014533_R-gene_LecRK
XLOC_014583_R-gene_LecRK
XLOC_046525_R-gene_LecRK
XLOC_009176_R-gene_RLK
XLOC_023070_R-gene_dirigent-like
XLOC_035776_R-gene_RLK
XLOC_030874_R-gene_RLK
XLOC_029494_R-gene_LecRK
XLOC_007624_R-gene_LecRK
XLOC_015563_R-gene_RLK
XLOC_015872_R-gene_RLP
XLOC_041208_R-gene_RLK
XLOC_033582_R-gene_LecRK
XLOC_035914_R-gene_RLK
XLOC_048339_R-gene_dirigent-like
(B)
-0.2715807
0.36420965
1.0
-4.0
0.0
4.0
Day 2
Day 4
Day 8
Day 18
XLOC_036821_calcium mitochondrial uniporter
XLOC_040994_calcium-binding ef-hand family protein
XLOC_047850_calcium mitochondrial uniporter
XLOC_006110_calcium-dependent lipid binding
XLOC_007396_calcium-binding ef-hand family protein
XLOC_025449_calcium mitochondrial uniporter
XLOC_048025_calcium-binding ef-hand family protein
XLOC_047408_calcium-binding ef-hand family protein
XLOC_019174_calcium-binding ef-hand family protein
XLOC_025337_calcium voltage-dependent channel
XLOC_020815_calcium-dependent phosphodiesterase
XLOC_036940_calcium-binding ef-hand family protein
XLOC_005174_calcineurin b-like protein
XLOC_047054_calcium-dependent phosphodiesterase
(C)
-0.112333536
0.44383323
1.0
-4.0
0.0
4.0
Day 2
Day 4
Day 8
Day 18
XLOC_009776_phosphatase 2c
XLOC_017996_CBL-interacting kinase
XLOC_038945_CBL-interacting kinase
XLOC_039506_OXI1-kinase
XLOC_045757_WNK-kinase
XLOC_026042_MAP-kinase
XLOC_030154_CBL-interacting kinase
XLOC_025319_CBL-interacting kinase
XLOC_010902_tyrosine phosphatase
XLOC_038563_kinase-interacting protein
XLOC_010980_MAP-kinase
XLOC_024486_tyrosine kinase
(D)
-0.4509666
0.2745167
1.0
-4.0
0.0
4.0
Day 2
Day 4
Day 8
Day 18
XLOC_030137_WRKY
XLOC_027037_IAA-induced
XLOC_018678_TCP
XLOC_014620_k-box and MADS box
XLOC_046912_MYB domain
XLOC_012526_C2H2 zinc finger
XLOC_020427_b-box zinc fingeer
XLOC_024107_WRKY
XLOC_009779_LOB domain
XLOC_016481_bHLH
XLOC_002794_bHLH
XLOC_027979_AP2/ERF_ERF
XLOC_040893_NAC domain
XLOC_006785_NAC domain
XLOC_011028_bHLH
XLOC_039651_AP2/ERF_ERF
XLOC_041305_NAC domain
XLOC_014625_WRKY
XLOC_039831_MYB domain
XLOC_015127_bZIP
XLOC_011244_bZIP
XLOC_017258_MYB domain
XLOC_030932_C2H2 zinc finger
XLOC_039980_NAC domain
XLOC_033878_NAC domain
XLOC_013230_WRKY
XLOC_016814_homeobox-leucine zipper
XLOC_005021_AP2/ERF_ERF
XLOC_025002_NAC domain
XLOC_010023_NAC domain
XLOC_046391_WRKY
XLOC_019924_bHLH
XLOC_049949_bZIP
XLOC_014539_GRAS
XLOC_041622_NAC domain
XLOC_016754_MYB domain
XLOC_014621_k-box and MADS box
XLOC_030435_winged-helix
XLOC_025350_bHLH
XLOC_016285_NAC domain
XLOC_030337_C2H2 zinc finger
XLOC_015378_MYB domain
XLOC_020440_WRKY
XLOC_031817_NAC domain
XLOC_041283_MYB domain
XLOC_027945_DOF zinc finger
XLOC_036585_TCP
XLOC_010519_MYB domain
XLOC_043905_C2H2 zinc finger
XLOC_033192_C2H2 zinc finger
XLOC_031296_LOB domain
XLOC_011383_MYB domain
XLOC_040045_MYB domain
XLOC_027114_WRKY
XLOC_029562_KOW domain
XLOC_007933_winged-helix
XLOC_017821_WRKY
XLOC_010407_WRKY
XLOC_047726_WRKY
XLOC_020250_NAC domain
XLOC_005023_AP2/ERF_ERF
XLOC_021742_NAC domain
XLOC_036889_GRAS
XLOC_001887_LOB domain
XLOC_048317_NAC domain
XLOC_030276_global-b1
XLOC_031480_MYB domain
XLOC_005557_homeobox-leucine zipper
XLOC_012820_WRKY
XLOC_016880_C2H2 zinc finger
XLOC_009090_MYB domain
XLOC_021564_WRKY
XLOC_034093_WRKY
XLOC_008807_GRAS
XLOC_008126_AP2/ERF_DREB
XLOC_024419_WRKY

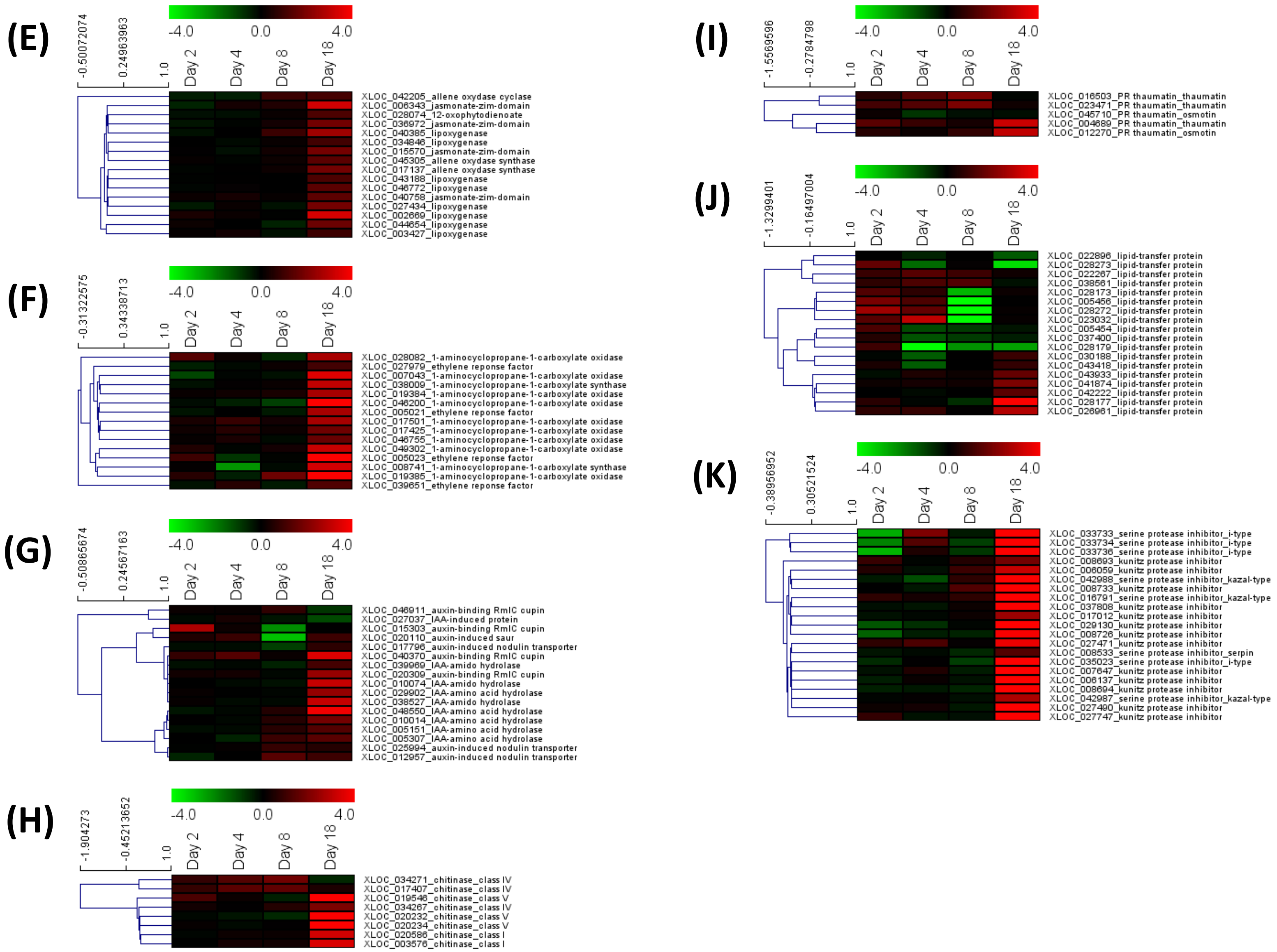

(E)
-4.0 0.0 4.0
Day 2 Day 4 Day 8 Day 18
-0.50072074 0.24963963 1.0
XLOC_042205_allene oxydase cyclase
XLOC_006343_jasmonate-zim-domain
XLOC_028074_12-oxophytodienoate
XLOC_036972_jasmonate-zim-domain
XLOC_040385_lipoxygenase
XLOC_034846_lipoxygenase
XLOC_015570_jasmonate-zim-domain
XLOC_045305_allene oxydase synthase
XLOC_017137_allene oxydase synthase
XLOC_043188_lipoxygenase
XLOC_046772_lipoxygenase
XLOC_040758_jasmonate-zim-domain
XLOC_027434_lipoxygenase
XLOC_002669_lipoxygenase
XLOC_044654_lipoxygenase
XLOC_003427_lipoxygenase
(F)
-4.0 0.0 4.0
Day 2 Day 4 Day 8 Day 18
-0.31322575 0.34338713 1.0
XLOC_028082_1-aminocyclopropane-1-carboxylate oxidase
XLOC_027979_ethylene reponse factor
XLOC_007043_1-aminocyclopropane-1-carboxylate oxidase
XLOC_038009_1-aminocyclopropane-1-carboxylate synthase
XLOC_019384_1-aminocyclopropane-1-carboxylate oxidase
XLOC_046200_1-aminocyclopropane-1-carboxylate oxidase
XLOC_005021_ethylene reponse factor
XLOC_017501_1-aminocyclopropane-1-carboxylate oxidase
XLOC_017425_1-aminocyclopropane-1-carboxylate oxidase
XLOC_046755_1-aminocyclopropane-1-carboxylate oxidase
XLOC_049302_1-aminocyclopropane-1-carboxylate oxidase
XLOC_005023_ethylene reponse factor
XLOC_008741_1-aminocyclopropane-1-carboxylate synthase
XLOC_019385_1-aminocyclopropane-1-carboxylate oxidase
XLOC_039651_ethylene reponse factor
(G)
-4.0 0.0 4.0
Day 2 Day 4 Day 8 Day 18
-0.50865674 0.24567163 1.0
XLOC_046911_auxin-binding RmlC cupin
XLOC_027037_IAA-induced protein
XLOC_015303_auxin-binding RmlC cupin
XLOC_020110_auxin-induced saur
XLOC_017796_auxin-induced nodulin transporter
XLOC_040370_auxin-binding RmlC cupin
XLOC_039969_IAA-amido hydrolase
XLOC_020309_auxin-binding RmlC cupin
XLOC_010074_IAA-amido hydrolase
XLOC_029902_IAA-amino acid hydrolase
XLOC_038527_IAA-amido hydrolase
XLOC_048550_IAA-amino acid hydrolase
XLOC_010014_IAA-amino acid hydrolase
XLOC_005151_IAA-amino acid hydrolase
XLOC_005307_IAA-amino acid hydrolase
XLOC_025994_auxin-induced nodulin transporter
XLOC_012957_auxin-induced nodulin transporter
(H)
-4.0 0.0 4.0
Day 2 Day 4 Day 8 Day 18
-1.904273 -0.4521365 1.0
XLOC_034271_chitinase_class IV
XLOC_017407_chitinase_class IV
XLOC_019546_chitinase_class V
XLOC_034267_chitinase_class IV
XLOC_020232_chitinase_class V
XLOC_020234_chitinase_class V
XLOC_020586_chitinase_class I
XLOC_003576_chitinase_class I
(I)
-4.0 0.0 4.0
Day 2 Day 4 Day 8 Day 18
-1.5569596 -0.2784798 1.0
XLOC_016503_PR thaumatin_thaumatin
XLOC_023471_PR thaumatin_thaumatin
XLOC_045710_PR thaumatin_osmotin
XLOC_004689_PR thaumatin_thaumatin
XLOC_012270_PR thaumatin_osmotin
(J)
-4.0 0.0 4.0
Day 2 Day 4 Day 8 Day 18
-1.3299401 -0.16497004 1.0
XLOC_022896_lipid-transfer protein
XLOC_028273_lipid-transfer protein
XLOC_022267_lipid-transfer protein
XLOC_038561_lipid-transfer protein
XLOC_028173_lipid-transfer protein
XLOC_005456_lipid-transfer protein
XLOC_028272_lipid-transfer protein
XLOC_023032_lipid-transfer protein
XLOC_005454_lipid-transfer protein
XLOC_037400_lipid-transfer protein
XLOC_028179_lipid-transfer protein
XLOC_030188_lipid-transfer protein
XLOC_043418_lipid-transfer protein
XLOC_043933_lipid-transfer protein
XLOC_041874_lipid-transfer protein
XLOC_042222_lipid-transfer protein
XLOC_028177_lipid-transfer protein
XLOC_026961_lipid-transfer protein
(K)
-4.0 0.0 4.0
Day 2 Day 4 Day 8 Day 18
-0.38956952 0.30521524 1.0
XLOC_033733_serine protease inhibitor_i-type
XLOC_033734_serine protease inhibitor_i-type
XLOC_033736_serine protease inhibitor_i-type
XLOC_008693_kunitz protease inhibitor
XLOC_006059_kunitz protease inhibitor
XLOC_042988_serine protease inhibitor_kazal-type
XLOC_008733_kunitz protease inhibitor
XLOC_016791_serine protease inhibitor_kazal-type
XLOC_037808_kunitz protease inhibitor
XLOC_017012_kunitz protease inhibitor
XLOC_029130_kunitz protease inhibitor
XLOC_008726_kunitz protease inhibitor
XLOC_027471_kunitz protease inhibitor
XLOC_008533_serine protease inhibitor_serpin
XLOC_035023_serine protease inhibitor_i-type
XLOC_007647_kunitz protease inhibitor
XLOC_006137_kunitz protease inhibitor
XLOC_008694_kunitz protease inhibitor
XLOC_042987_serine protease inhibitor_kazal-type
XLOC_027490_kunitz protease inhibitor
XLOC_027747_kunitz protease inhibitor

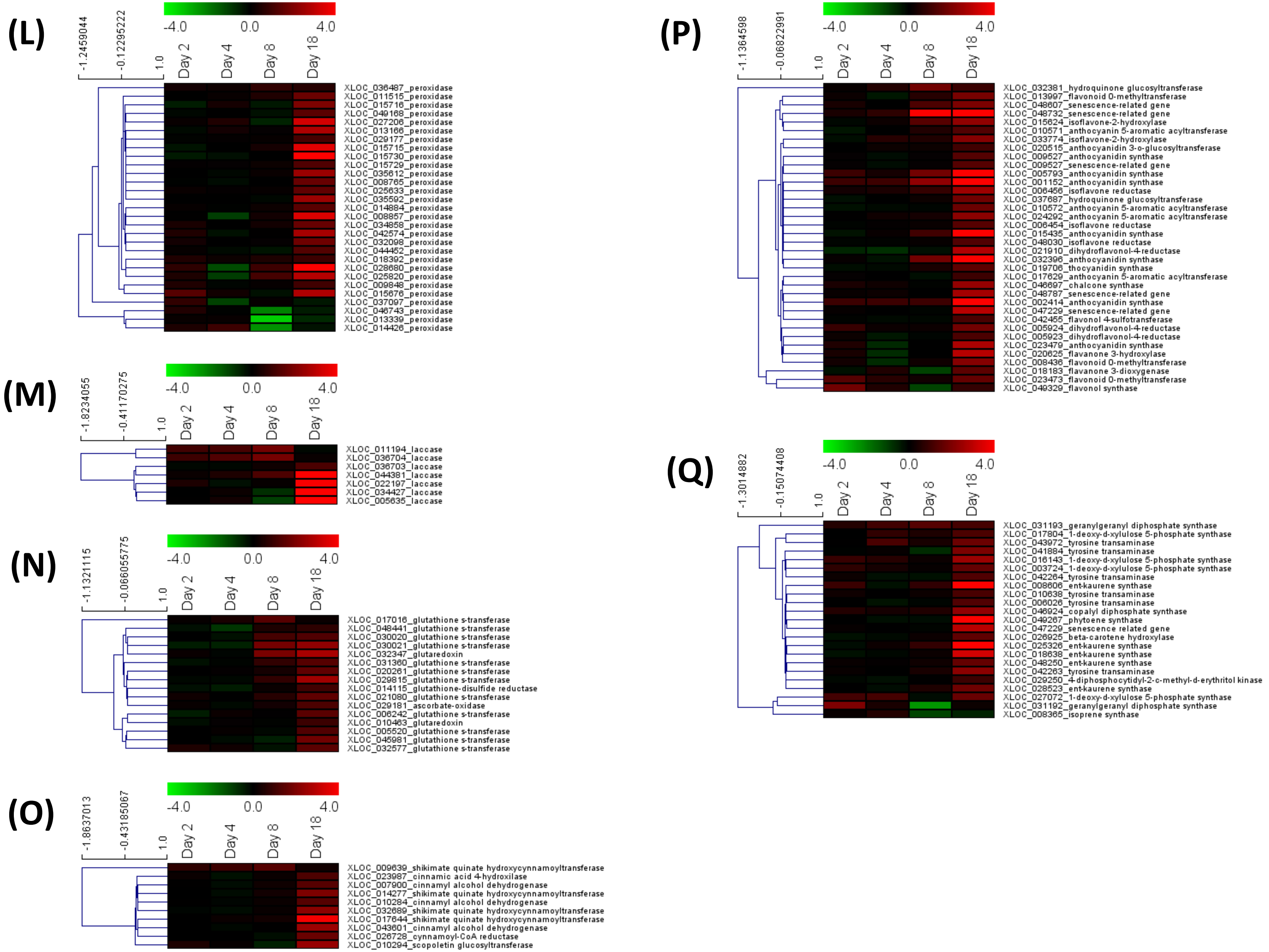
(L)
-1.2459044
-0.12295222
1.0
-4.0
0.0
4.0
Day 2
Day 4
Day 8
Day 18
XLOC_036487_peroxidase
XLOC_011515_peroxidase
XLOC_015716_peroxidase
XLOC_049168_peroxidase
XLOC_027206_peroxidase
XLOC_013166_peroxidase
XLOC_029177_peroxidase
XLOC_015715_peroxidase
XLOC_015730_peroxidase
XLOC_015729_peroxidase
XLOC_035612_peroxidase
XLOC_008765_peroxidase
XLOC_025633_peroxidase
XLOC_035592_peroxidase
XLOC_014884_peroxidase
XLOC_008857_peroxidase
XLOC_034858_peroxidase
XLOC_042574_peroxidase
XLOC_032098_peroxidase
XLOC_044452_peroxidase
XLOC_018392_peroxidase
XLOC_028680_peroxidase
XLOC_025820_peroxidase
XLOC_009848_peroxidase
XLOC_015676_peroxidase
XLOC_037097_peroxidase
XLOC_046743_peroxidase
XLOC_013339_peroxidase
XLOC_014426_peroxidase
(M)
-1.8234055
-0.41170275
1.0
-4.0
0.0
4.0
Day 2
Day 4
Day 8
Day 18
XLOC_011194_laccase
XLOC_036704_laccase
XLOC_036703_laccase
XLOC_044381_laccase
XLOC_022197_laccase
XLOC_034427_laccase
XLOC_005635_laccase
(N)
-1.1321115
-0.066055775
1.0
-4.0
0.0
4.0
Day 2
Day 4
Day 8
Day 18
XLOC_017016_glutathione s-transferase
XLOC_048441_glutathione s-transferase
XLOC_030020_glutathione s-transferase
XLOC_030021_glutathione s-transferase
XLOC_032347_glutaredoxin
XLOC_031360_glutathione s-transferase
XLOC_020261_glutathione s-transferase
XLOC_029815_glutathione s-transferase
XLOC_014115_glutathione-disulfide reductase
XLOC_021080_glutathione s-transferase
XLOC_029181_ascorbate-oxidase
XLOC_006242_glutathione s-transferase
XLOC_010463_glutaredoxin
XLOC_005520_glutathione s-transferase
XLOC_045981_glutathione s-transferase
XLOC_032577_glutathione s-transferase
(O)
-1.8637013
-0.43185067
1.0
-4.0
0.0
4.0
Day 2
Day 4
Day 8
Day 18
XLOC_009639_shikimate quinate hydroxycynnamoyltransferase
XLOC_023987_cinnamic acid 4-hydroxilase
XLOC_007900_cinnamyl alcohol dehydrogenase
XLOC_014277_shikimate quinate hydroxycynnamoyltransferase
XLOC_010284_cinnamyl alcohol dehydrogenase
XLOC_032689_shikimate quinate hydroxycynnamoyltransferase
XLOC_017644_shikimate quinate hydroxycynnamoyltransferase
XLOC_043601_cinnamyl alcohol dehydrogenase
XLOC_026728_cynnamoyl-CoA reductase
XLOC_010294_scopoletin glucosyltransferase
(P)
-1.1364598
-0.06822991
1.0
-4.0
0.0
4.0
Day 2
Day 4
Day 8
Day 18
XLOC_032381_hydroquinone glucosyltransferase
XLOC_013997_flavonoid 0-methyltransferase
XLOC_048607_senescence-related gene
XLOC_048732_senescence-related gene
XLOC_015624_isoflavone-2-hydroxylase
XLOC_010571_anthocyanin 5-aromatic acyltransferase
XLOC_033774_isoflavone-2-hydroxylase
XLOC_020515_anthocyanidin 3-o-glucosyltransferase
XLOC_009527_anthocyanidin synthase
XLOC_009527_senescence-related gene
XLOC_005793_anthocyanidin synthase
XLOC_001152_anthocyanidin synthase
XLOC_006456_isoflavone reductase
XLOC_037687_hydroquinone glucosyltransferase
XLOC_010572_anthocyanin 5-aromatic acyltransferase
XLOC_024292_anthocyanin 5-aromatic acyltransferase
XLOC_006454_isoflavone reductase
XLOC_015435_anthocyanidin synthase
XLOC_048030_isoflavone reductase
XLOC_021910_dihydroflavonol-4-reductase
XLOC_032396_anthocyanidin synthase
XLOC_019706_thocyanidin synthase
XLOC_017629_anthocyanin 5-aromatic acyltransferase
XLOC_046697_chalcone synthase
XLOC_048787_senescence-related gene
XLOC_002414_anthocyanidin synthase
XLOC_047229_senescence-related gene
XLOC_042455_flavonol 4-sulfotransferase
XLOC_005924_dihydroflavonol-4-reductase
XLOC_005923_dihydroflavonol-4-reductase
XLOC_023479_anthocyanidin synthase
XLOC_020625_flavanone 3-hydroxylase
XLOC_008436_flavonoid 0-methyltransferase
XLOC_018183_flavanone 3-dioxygenase
XLOC_023473_flavonoid 0-methyltransferase
XLOC_049329_flavonol synthase
(Q)
-1.3014882
-0.15074408
1.0
-4.0
0.0
4.0
Day 2
Day 4
Day 8
Day 18
XLOC_031193_geranylgeranyl diphosphate synthase
XLOC_017804_1-deoxy-d-xylulose 5-phosphate synthase
XLOC_043972_tyrosine transaminase
XLOC_041884_tyrosine transaminase
XLOC_016143_1-deoxy-d-xylulose 5-phosphate synthase
XLOC_003724_1-deoxy-d-xylulose 5-phosphate synthase
XLOC_042264_tyrosine transaminase
XLOC_008606_ent-kaurene synthase
XLOC_010638_tyrosine transaminase
XLOC_006026_tyrosine transaminase
XLOC_046924_copalyl diphosphate synthase
XLOC_049267_phytoene synthase
XLOC_047229_senescence related gene
XLOC_026925_beta-carotene hydroxylase
XLOC_025326_ent-kaurene synthase
XLOC_018638_ent-kaurene synthase
XLOC_048250_ent-kaurene synthase
XLOC_042263_tyrosine transaminase
XLOC_029250_4-diphosphocytidyl-2-c-methyl-d-erythritol kinase
XLOC_028523_ent-kaurene synthase
XLOC_027072_1-deoxy-d-xylulose 5-phosphate synthase
XLOC_031192_geranylgeranyl diphosphate synthase
XLOC_008365_isoprene synthase

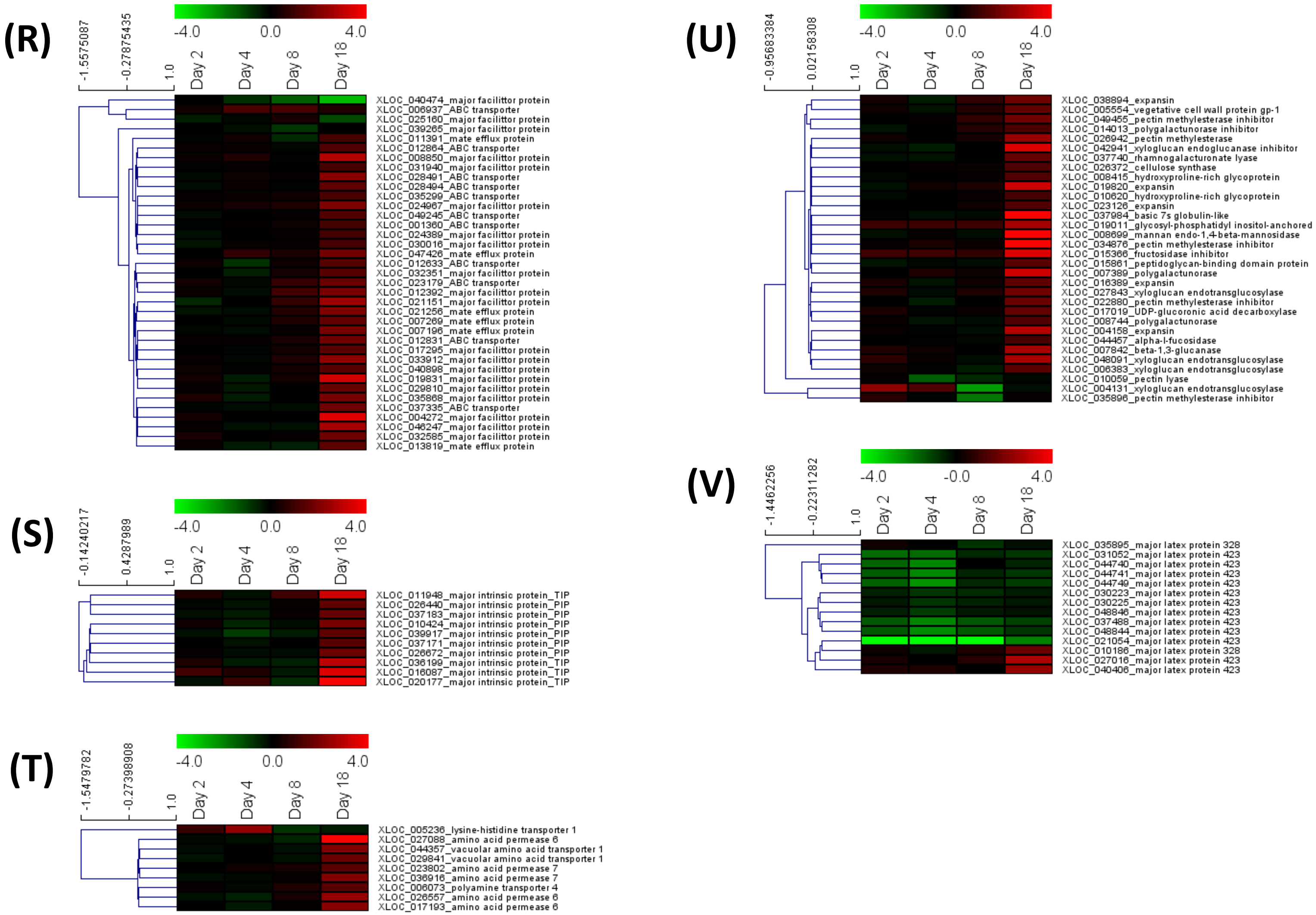

(R)
-1.5575087
-0.27875435
1.0
-4.0
0.0
4.0
Day 2
Day 4
Day 8
Day 18
XLOC_040474_major facilittor protein
XLOC_006937_ABC transporter
XLOC_025160_major facilittor protein
XLOC_039265_major facilittor protein
XLOC_011391_mate efflux protein
XLOC_012864_ABC transporter
XLOC_008850_major facilittor protein
XLOC_031940_major facilittor protein
XLOC_028491_ABC transporter
XLOC_028494_ABC transporter
XLOC_035299_ABC transporter
XLOC_024967_major facilittor protein
XLOC_049245_ABC transporter
XLOC_001360_ABC transporter
XLOC_024389_major facilittor protein
XLOC_030016_major facilittor protein
XLOC_047426_mate efflux protein
XLOC_012633_ABC transporter
XLOC_032351_major facilittor protein
XLOC_023179_ABC transporter
XLOC_012392_major facilittor protein
XLOC_021151_major facilittor protein
XLOC_021256_mate efflux protein
XLOC_007269_mate efflux protein
XLOC_007196_mate efflux protein
XLOC_012831_ABC transporter
XLOC_017295_major facilittor protein
XLOC_033912_major facilittor protein
XLOC_040898_major facilittor protein
XLOC_019831_major facilittor protein
XLOC_029810_major facilittor protein
XLOC_035868_major facilittor protein
XLOC_037335_ABC transporter
XLOC_004272_major facilittor protein
XLOC_046247_major facilittor protein
XLOC_032585_major facilittor protein
XLOC_013819_mate efflux protein
(S)
-0.14240217
0.4287989
1.0
-4.0
0.0
4.0
Day 2
Day 4
Day 8
Day 18
XLOC_011948_major intrinsic protein_TIP
XLOC_026440_major intrinsic protein_PIP
XLOC_037183_major intrinsic protein_PIP
XLOC_010424_major intrinsic protein_PIP
XLOC_039917_major intrinsic protein_PIP
XLOC_037171_major intrinsic protein_PIP
XLOC_026672_major intrinsic protein_PIP
XLOC_036199_major intrinsic protein_TIP
XLOC_016087_major intrinsic protein_TIP
XLOC_020177_major intrinsic protein_TIP
(T)
-1.5479782
-0.27398908
1.0
-4.0
0.0
4.0
Day 2
Day 4
Day 8
Day 18
XLOC_005236_lysine-histidine transporter 1
XLOC_027088_amino acid permease 6
XLOC_044357_vacuolar amino acid transporter 1
XLOC_029841_vacuolar amino acid transporter 1
XLOC_023802_amino acid permease 7
XLOC_036916_amino acid permease 7
XLOC_006073_polyamine transporter 4
XLOC_026557_amino acid permease 6
XLOC_017193_amino acid permease 6
(U)
-0.95683384
0.02158308
1.0
-4.0
0.0
4.0
Day 2
Day 4
Day 8
Day 18
XLOC_038894_expansin
XLOC_005554_vegetative cell wall protein gp-1
XLOC_049455_pectin methylesterase inhibitor
XLOC_014013_polygalacturonase inhibitor
XLOC_026942_pectin methylesterase
XLOC_042941_xyloglucan endoglucanase inhibitor
XLOC_037740_rhamnogalacturonate lyase
XLOC_026372_cellulose synthase
XLOC_008415_hydroxyproline-rich glycoprotein
XLOC_019820_expansin
XLOC_010620_hydroxyproline-rich glycoprotein
XLOC_023126_expansin
XLOC_037984_basic 7s globulin-like
XLOC_019011_glycosyl-phosphatidyl inositol-anchored
XLOC_008699_mannan endo-1,4-beta-mannosidase
XLOC_034876_pectin methylesterase inhibitor
XLOC_015366_fructosidase inhibitor
XLOC_015861_peptidoglycan-binding domain protein
XLOC_007389_polygalacturonase
XLOC_016389_expansin
XLOC_027843_xyloglucan endotransglucosylase
XLOC_022880_pectin methylesterase inhibitor
XLOC_017019_UDP-glucoronic acid decarboxylase
XLOC_008744_polygalacturonase
XLOC_004158_expansin
XLOC_044457_alpha-l-fucosidase
XLOC_007842_beta-1,3-glucanase
XLOC_048091_xyloglucan endotransglucosylase
XLOC_006383_xyloglucan endotransglucosylase
XLOC_010059_pectin lyase
XLOC_004131_xyloglucan endotransglucosylase
XLOC_035896_pectin methylesterase inhibitor
(V)
-1.4462256
-0.22311282
1.0
-4.0
-0.0
4.0
Day 2
Day 4
Day 8
Day 18
XLOC_035895_major latex protein 328
XLOC_031052_major latex protein 423
XLOC_044740_major latex protein 423
XLOC_044741_major latex protein 423
XLOC_044749_major latex protein 423
XLOC_030223_major latex protein 423
XLOC_030225_major latex protein 423
XLOC_048846_major latex protein 423
XLOC_037488_major latex protein 423
XLOC_048844_major latex protein 423
XLOC_021054_major latex protein 423
XLOC_010186_major latex protein 328
XLOC_027016_major latex protein 423
XLOC_040406_major latex protein 423

Figure S5. Expression patterns of major gene groups in flax through the time course upon inoculation with *F. oxysporum* f. sp. *lini*. Genes depicted are significantly differentially expressed at least at one time point (*q* = 0.05). **(A)** Signal perception genes. **(B)** Calcium-related genes. **(C)** Kinases. **(D)** Transcription factors. **(E)** Jasmonate-related. **(F)** Ethylene-related. **(G)** Auxin-related. **(H)** Chitinases. **(I)** Thaumatins. **(J)** Lipid transfer proteins. **(K)** Protease inhibitors. **(L)** Peroxidases. **(M)** Laccases. **(N)** Gluthatione-related. **(O)** Phenylpropanoid metabolism. **(P)** Flavonoid metabolism. **(Q)** Isoprenoid metabolism. **(R)** Transporters. **(S)** Major intrinsic proteins. **(T)** Amino acid permeases. **(U)** Cell wall. **(V)** Major latex proteins.
