# Supplementary material for: RNA-seq Transcriptome Response of Flax (Linum usitatissimum L.) to the Pathogenic Fungus Fusarium oxysporum f. sp. lini
Source: Front Plant Sci. 2016 Nov 24;7:1766. doi: 10.3389/fpls.2016.01766 (PMC5121121; doi:10.3389/fpls.2016.01766)
Supplement: Supplementary file 9 [file Image_6.PDF]

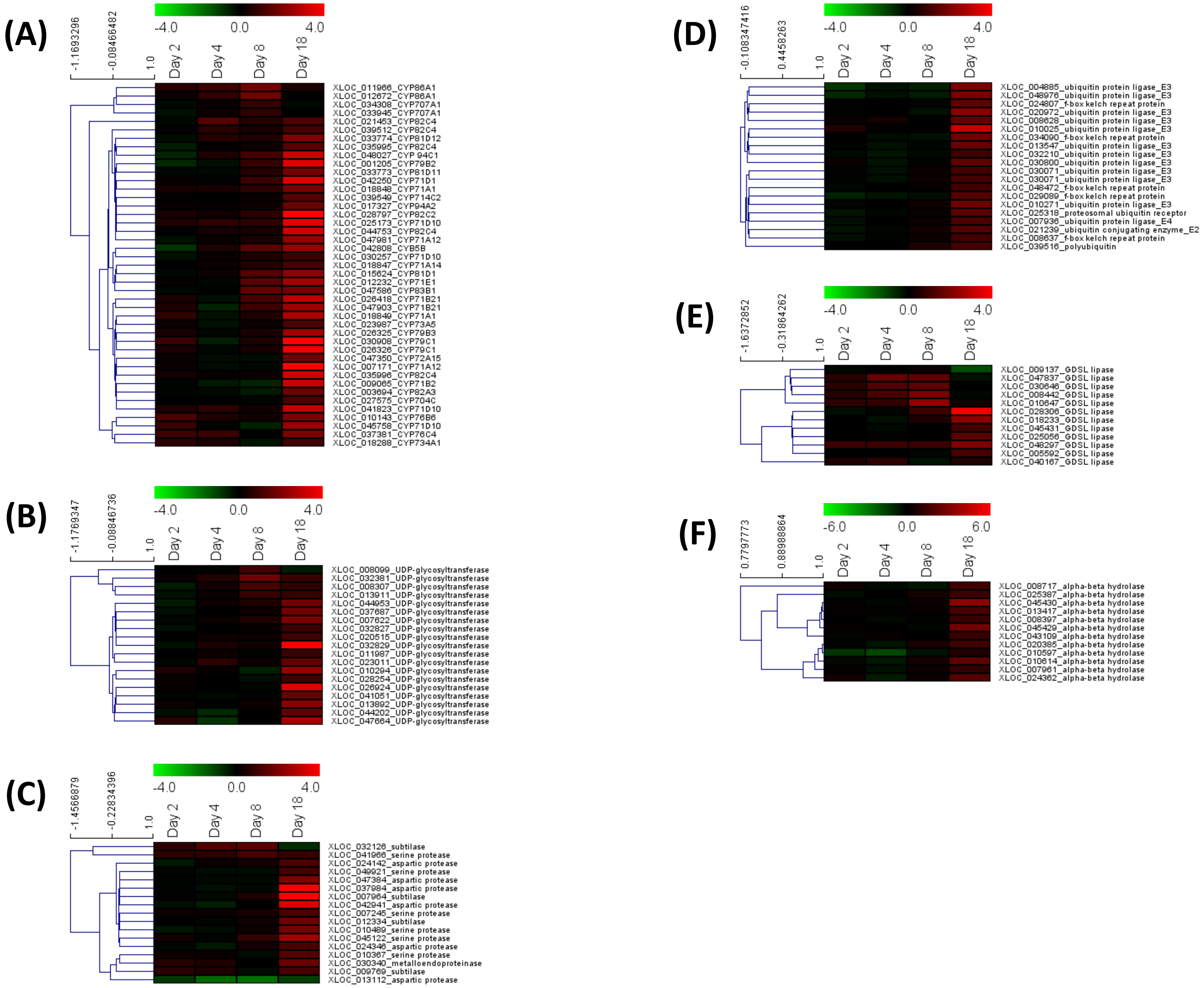

(A)
-4.0
0.0
4.0
-1.1693296
-0.08466482
1.0
Day 2
Day 4
Day 8
Day 18
XLOC_011966_CYP86A1
XLOC_012672_CYP86A1
XLOC_034308_CYP707A1
XLOC_033945_CYP707A1
XLOC_021453_CYP82C4
XLOC_039512_CYP82C4
XLOC_033774_CYP81D12
XLOC_035995_CYP82C4
XLOC_048027_CYP 94C1
XLOC_001205_CYP79B2
XLOC_033773_CYP81D11
XLOC_042250_CYP71D1
XLOC_018848_CYP71A1
XLOC_039549_CYP714C2
XLOC_017327_CYP94A2
XLOC_028797_CYP82C2
XLOC_025173_CYP71D10
XLOC_044753_CYP82C4
XLOC_047981_CYP71A12
XLOC_042808_CYB5B
XLOC_030257_CYP71D10
XLOC_018847_CYP71A14
XLOC_015624_CYP81D1
XLOC_012232_CYP71E1
XLOC_047586_CYP83B1
XLOC_026418_CYP71B21
XLOC_047903_CYP71B21
XLOC_018849_CYP71A1
XLOC_023987_CYP73A5
XLOC_026325_CYP79B3
XLOC_030908_CYP79C1
XLOC_026326_CYP79C1
XLOC_047350_CYP72A15
XLOC_007171_CYP71A12
XLOC_035996_CYP82C4
XLOC_009065_CYP71B2
XLOC_003694_CYP82A3
XLOC_027575_CYP704C
XLOC_041823_CYP71D10
XLOC_010143_CYP76B6
XLOC_045758_CYP71D10
XLOC_037381_CYP76C4
XLOC_018288_CYP734A1
(B)
-4.0
0.0
4.0
-1.1769347
-0.08846736
1.0
Day 2
Day 4
Day 8
Day 18
XLOC_008099_UDP-glycosyltransferase
XLOC_032381_UDP-glycosyltransferase
XLOC_008307_UDP-glycosyltransferase
XLOC_013911_UDP-glycosyltransferase
XLOC_044953_UDP-glycosyltransferase
XLOC_037687_UDP-glycosyltransferase
XLOC_007622_UDP-glycosyltransferase
XLOC_032827_UDP-glycosyltransferase
XLOC_020515_UDP-glycosyltransferase
XLOC_032829_UDP-glycosyltransferase
XLOC_011987_UDP-glycosyltransferase
XLOC_023011_UDP-glycosyltransferase
XLOC_010294_UDP-glycosyltransferase
XLOC_028254_UDP-glycosyltransferase
XLOC_026924_UDP-glycosyltransferase
XLOC_041051_UDP-glycosyltransferase
XLOC_013892_UDP-glycosyltransferase
XLOC_044202_UDP-glycosyltransferase
XLOC_047664_UDP-glycosyltransferase
(C)
-4.0
0.0
4.0
-1.4566879
-0.22834396
1.0
Day 2
Day 4
Day 8
Day 18
XLOC_032126_subtilase
XLOC_041966_serine protease
XLOC_024142_aspartic protease
XLOC_049921_serine protease
XLOC_047384_aspartic protease
XLOC_037984_aspartic protease
XLOC_007964_subtilase
XLOC_042941_aspartic protease
XLOC_007245_serine protease
XLOC_012334_subtilase
XLOC_010489_serine protease
XLOC_045122_serine protease
XLOC_024346_aspartic protease
XLOC_010367_serine protease
XLOC_030340_metalloendoproteinase
XLOC_009769_subtilase
XLOC_013112_aspartic protease
(D)
-4.0
0.0
4.0
-0.108347416
0.4458263
1.0
Day 2
Day 4
Day 8
Day 18
XLOC_004885_ubiquitin protein ligase_E3
XLOC_048976_ubiquitin protein ligase_E3
XLOC_024807_f-box kelch repeat protein
XLOC_020972_ubiquitin protein ligase_E3
XLOC_008628_ubiquitin protein ligase_E3
XLOC_010025_ubiquitin protein ligase_E3
XLOC_034090_f-box kelch repeat protein
XLOC_013547_ubiquitin protein ligase_E3
XLOC_032210_ubiquitin protein ligase_E3
XLOC_030800_ubiquitin protein ligase_E3
XLOC_030071_ubiquitin protein ligase_E3
XLOC_030071_ubiquitin protein ligase_E3
XLOC_048472_f-box kelch repeat protein
XLOC_029089_f-box kelch repeat protein
XLOC_010271_ubiquitin protein ligase_E3
XLOC_025318_proteosomal ubiquitin receptor
XLOC_007936_ubiquitin protein ligase_E4
XLOC_021239_ubiquitin conjugating enzyme_E2
XLOC_008637_f-box kelch repeat protein
XLOC_039516_polyubiquitin
(E)
-4.0
0.0
4.0
-1.6372852
-0.31864262
1.0
Day 2
Day 4
Day 8
Day 18
XLOC_009137_GDSL lipase
XLOC_047837_GDSL lipase
XLOC_030646_GDSL lipase
XLOC_008442_GDSL lipase
XLOC_010647_GDSL lipase
XLOC_028306_GDSL lipase
XLOC_018233_GDSL lipase
XLOC_045431_GDSL lipase
XLOC_025056_GDSL lipase
XLOC_048297_GDSL lipase
XLOC_005592_GDSL lipase
XLOC_040167_GDSL lipase
(F)
-6.0
0.0
6.0
0.7797773
0.88988864
1.0
Day 2
Day 4
Day 8
Day 18
XLOC_008717_alpha-beta hydrolase
XLOC_025387_alpha-beta hydrolase
XLOC_045430_alpha-beta hydrolase
XLOC_013417_alpha-beta hydrolase
XLOC_008397_alpha-beta hydrolase
XLOC_045429_alpha-beta hydrolase
XLOC_043109_alpha-beta hydrolase
XLOC_020385_alpha-beta hydrolase
XLOC_010597_alpha-beta hydrolase
XLOC_010614_alpha-beta hydrolase
XLOC_007961_alpha-beta hydrolase
XLOC_024362_alpha-beta hydrolase

Figure S6. Expression patterns of other relevant gene groups in flax through the time course upon inoculation with *F. oxysporum* f. sp. *lini*. Genes depicted are significantly differentially expressed at least at one time point *(q* = 0.05). **(A)** Cytochrome P genes. **(B)**. UDP glucosyltransferases. **(C)** Protein degradation. **(D)** Proteosome/ubiquitin-related. **(E)** GDSL lipases. **(F)** Alpha/beta hydrolases.
